# Association of Polygenic Risk for Psychiatric Disorders with Cardiometabolic Disease

**DOI:** 10.1101/2025.03.11.25323757

**Published:** 2025-03-11

**Authors:** Jacob Bergstedt, Kadri Kõiv, Andreas Jangmo, Marit Haram, Piotr P. Jaholkowski, Jorien L. Treur, Isabell Brikell, Zheng Chang, Henrik Larsson, Patrik K. E. Magnusson, Andrew M. McIntosh, Cathryn M. Lewis, Brian K. Lee, Ida E. Sønderby, Yi Lu, Patrick F. Sullivan, Unnur A. Valdimarsdóttir, Estonian Biobank Research Team, Ole Andreassen, Martin Tesli, Kelli Lehto, Fang Fang

**Affiliations:** 1 Unit of Integrative Epidemiology, Institute of Environmental Medicine, Karolinska Institutet, Stockholm, Sweden; 2 Estonian Genome Centre, Institute of Genomics, University of Tartu, Tartu, Estonia; 3 Department of Mental Health and Suicide, Norwegian Institute of Public Health, Oslo, Norway; 4 Division of Mental Health and Addiction, Oslo University Hospital, Oslo, Norway; 5 Institute of Clinical Medicine, University of Oslo, Oslo, Norway; 6 Center for Precision Psychiatry, University of Oslo, Oslo, Norway; 7 Genetic Epidemiology, Department of Psychiatry, Amsterdam UMC, University of Amsterdam, Amsterdam, Netherlands; 8 Department of Medical Epidemiology and Biostatistics, Karolinska Institutet, Stockholm, Sweden; 9 Department of Global Public Health and Primary Care, University of Bergen, Bergen, Norway; 10 Department of Biomedicine, Aarhus University, Aarhus, Denmark; 11 School of Medical Sciences, Faculty of Medicine and Health, Örebro University, Örebro, Sweden; 12 Centre for Clinical Brain Sciences, University of Edinburgh, Royal Edinburgh Hospital, Edinburgh, UK; 13 Centre for Genomics and Experimental Medicine, University of Edinburgh, Edinburgh, UK; 14 Social, Genetic and Developmental Psychiatry Centre, King’s College London, London, UK; 15 Department of Medical and Molecular Genetics, King’s College London, London, UK; 16 A.J. Drexel Autism Institute, Drexel University, Philadelphia, PA, USA; 17 Dornsife School of Public Health, Drexel University, Philadelphia, PA, USA; 18 Department of Psychiatry, University of North Carolina at Chapel Hill, Chapel Hill, NC, USA; 19 Department of Genetics, University of North Carolina at Chapel Hill, Chapel Hill, NC, USA; 20 Centre of Public Health Sciences, Faculty of Medicine, School of Health Sciences, University of Iceland, Reykjavik, Iceland; 21 Department of Epidemiology, Harvard TH Chan School of Public Health, Harvard University, Boston, MA, USA; 22 K.G. Jebsen Centre for Neurodevelopmental Disorders, University of Oslo and Oslo University Hospital, Oslo, Norway

## Abstract

**IMPORTANCE:**

Clinical diagnoses of psychiatric disorders are associated with cardiometabolic diseases (CMDs) such as type 2 diabetes and ischemic heart diseases. Studying how genetic liability for psychiatric disorders relate to CMD risk will offer novel insight into the relationship between psychiatric disorders and CMDs.

**OBJECTIVE:**

To evaluate the associations between psychiatric polygenic risk scores (PRSs) and clinically diagnosed CMDs while accounting for cross-disorder pleiotropy.

**DESIGN, SETTING, AND PARTICIPANTS:**

This study computed PRSs for attention deficit-hyperactivity disorder (ADHD), major depressive disorder (MDD), anxiety disorder, post-traumatic stress disorder (PTSD), bipolar disorder, and schizophrenia. The analysis was conducted in three population-based Northern European cohorts: the Swedish Twin Registry (STR, N=17,378 genotyped samples), the Estonian Biobank (EstBB, N=208,383), and the Norwegian Mother, Father and Child Cohort Study (MoBa, N=129,398). Associations between psychiatric PRSs and clinical diagnoses of 10 major CMDs (including metabolic diseases such as hyperlipidemia, obesity, and type 2 diabetes, and cardiovascular diseases such as hypertensive disease, arteriosclerosis, ischemic heart disease, heart failure, thromboembolic disease, cerebrovascular disease, and arrhythmias) were estimated using models that mutually adjusted for all psychiatric PRSs. Supplementary analyses were performed by additionally controlling for self-reported body mass index (BMI). A discordant twin-pair analysis was conducted in the STR (N=70,619) to assess the association between self-reported lifetime MDD and subsequent CMD risk while adjusting for familial factors shared between monozygotic and dizygotic co-twins.

**MAIN OUTCOMES AND MEASURES:**

Psychiatric PRSs were constructed based on both all available genetic risk variants and genome-wide significant risk variants from large-scale GWASs. Clinical diagnoses of psychiatric disorders and CMDs were ascertained through electronic health records (with primary care records used exclusively in the EstBB). Lifetime self-reported MDD in the STR was assessed via the Composite International Diagnostic Interview Short Form.

**RESULTS:**

PRSs for ADHD and MDD were associated with increased risk of all CMDs. The ADHD PRS showed stronger associations with metabolic disease, whereas the MDD PRS showed stronger associations with cardiovascular diseases. PRSs for anxiety disorder, PTSD, and bipolar disorder showed only limited associations with CMDs, while increased levels of schizophrenia PRSs were associated with decreased risk of CMDs. These associations remained after adjustment for BMI. Finally, twins endorsing lifetime MDD were found to have an increased risk of subsequent CMD diagnoses compared to their unexposed co-twins.

**CONCLUSIONS AND RELEVANCE:**

PRSs for ADHD and MDD showed robust associations with risk of CMDs and self-reported MDD was associated with subsequent CMD risk even after adjusting for familial factors shared between co-twins. These findings provide robust evidence for genetic overlap between ADHD and MDD with CMDs.

## Introduction

Cardiometabolic diseases (CMDs) substantially reduce life expectancy in individuals with psychiatric disorders^1–3^. Large-scale studies using electronic health records (EHRs) consistently show that most psychiatric diagnoses are linked to elevated CMD risk.^4,5^. Recent genome-wide association studies (GWASs) of psychiatric disorders have achieved sufficient statistical power to capture a substantial proportion of common variant genetic risk^6,7^, which has enabled the construction of polygenic risk scores (PRSs) —weighted sums of risk alleles based on GWAS effect sizes—to accurately capture genetic susceptibility^8^. Because PRSs are determined at birth, their associations with CMD outcomes are less prone to reverse causation and confounding than associations based on clinical diagnoses. PRS approaches have been employed to investigate relationships between CMDs and genetic liability to major depressive disorder (MDD)^9,10^, schizophrenia^9,10^, ADHD^11–13^, and post-traumatic stress disorder (PTSD)^14^. However, since psychiatric disorders exhibit substantial pleiotropy^15^, associations of psychiatric PRS might reflect polygenic overlap with other psychiatric disorders. To date, studies that examine associations of psychiatric PRS with CMDs while adjusting for other psychiatric PRSs remain lacking.

In this study, we leverage data from three large, population-based Northern European cohorts—the Swedish Twin Registry (STR), the Estonian Biobank (EstBB), and the Norwegian Mother, Father, and Child Cohort Study (MoBa)—comprising 355,159 genotyped individuals. Using longitudinal EHRs from both primary and specialist care, we assess the associations between PRSs for six psychiatric disorders (ADHD, MDD, anxiety disorder, PTSD, bipolar disorder, and schizophrenia) and clinical diagnoses of 10 major CMDs, including metabolic conditions (hyperlipidemia, obesity, and type 2 diabetes) as well as cardiovascular diseases (hypertensive disease, arteriosclerosis, ischemic heart disease, heart failure, thromboembolic disease, cerebrovascular disease, and arrhythmias). We explore independent associations by mutually adjusting for all psychiatric PRSs and, in an additional analysis, adjusting for self-reported body mass index (BMI). Finally, using questionnaire assessments of lifetime MDD in the STR, we perform a discordant twin analysis to further elucidate the relationship between MDD and CMDs.

## Methods

### Study participants

#### STR

We conducted PRS analysis in the Screening Across the Lifespan Twin (SALT) cohort of the STR. The SALT cohort is a population-based study of Swedish twins born between 1911 and 1958. When the data collection for SALT ended in 2002, 44,912 twins had participated^16^. The response rate was 65% for twins born 1911 to 1925 and 74% for twins born 1926 to 1958^17^. Data collection was conducted over the telephone by trained interviewers. Two studies nested in SALT were later conducted to measure genotypes. In the TwinGene study conducted between 2004 and 2008, 22,000 SALT participants born 1943 or earlier were invited to donate blood for genotyping with a response rate of 56%. In the SALTY study conducted between 2008 and 2010, 24,914 SALT participants born from 1943 to 1958 were invited to provide saliva sample for genotyping with a response rate of 47%^16^. After quality control preprocessing, the sample comprised 17,378 genotyped individuals (Table 1).

For the discordant twin analysis, we considered the SALT cohort and the Study of Twin Adults: Genes and Environment (STAGE) cohort. The STAGE cohort invited 42,582 twins born 1958–1985 to participate in a web-based survey, or if preferred, a phone interview with a trained interviewer with a response rate of 60%. The combined SALT and STAGE sample comprised 70,619 individuals.

The current study was approved by the Swedish Ethical Review Authority (registration numbers 2021–03197, 2021–02994, 2021–02994).

#### Estonian Biobank

The EstBB is a volunteer-based biobank with a sample size of approximately 210,000 participants, comprising around 20% of the adult population of Estonia^18–20^. The EstBB is regularly linked to national and regional EHR databases, such as the national quality registers, hospital records, the Estonian Health Insurance Fund (EHIF), and the National Health Information System, providing information on both primary and specialist care medical data.

#### MoBa

The Norwegian Mother, Father and Child Cohort Study (MoBa) is a population-based pregnancy cohort study conducted by the Norwegian Institute of Public Health. Participants were recruited from all over Norway from 1999–2008. The women consented to participation in 41% of the pregnancies. The cohort includes approximately 114,500 children, 95,200 mothers and 75,200 fathers. We only consider the parents in this study. Blood samples were obtained from both parents during pregnancy and from mothers and children (umbilical cord) at birth^21^. The establishment of MoBa and initial data collection was based on a license from the Norwegian Data Protection Agency and approval from The Regional Committees for Medical and Health Research Ethics. The MoBa cohort is currently regulated by the Norwegian Health Registry Act. The current study was approved by The Regional Committees for Medical and Health Research Ethics.

### PRS profiling

We leveraged large-scale GWASs of ADHD^22^, MDD^23^, anxiety disorder^24^, PTSD^25^, bipolar disorder^26^, and schizophrenia^7^ to compute PRSs in the STR, EstBB, and MoBa (eTable 1). Details on genotyping in STR, EstBB, and MoBa are given in the Supplement (eMethods). If the GWAS included samples from a cohort under study, we used leave-cohort-out GWAS summary statistics to compute PRSs. We removed 200 individuals diagnosed with schizophrenia in EstBB that were included in a seminal schizophrenia GWAS study that formed a basis for subsequent studies^27^. We computed PRSs using the SBayesR framework^28^. We used a map of linkage disequilibrium estimated from 2,865,810 SNPs in 50,000 UK Biobank samples using a shrinkage estimator^28^. In addition, we computed PRSs based solely on genome-wide significant variants using clumping and thresholding (*P*<5×10^−8^) implemented in PRSice-2^29^. This was done to perform a sensitivity analysis assessing associations less influenced by horizontal pleiotropy. For anxiety disorder and PTSD, PRSs based on genome-wide significant loci relied on very few variants (<4 across cohorts, eTable 2) and did not give reliable results. Results for those PRSs are therefore not shown. We scaled the PRSs using the standard deviation.

### Exposure and Outcome Measures

We ascertained psychiatric disorders and CMDs using EHR data. In the STR cohort, we used the 8^th^, 9^th^, and 10^th^ Swedish revisions of the International Classification of Diseases (ICD-8, 9, and 10) codes recorded in connection with an in-patient (national coverage from 1987) or out-patient (>80% national coverage since 2001) hospital visit in the Swedish Patient Register, or in connection with a death recorded in the Cause-of-Death Register. We considered both main and secondary diagnoses. One diagnosis was sufficient to be identified as a case. Diagnoses were recorded until the end of 2016.

In EstBB, we used ICD-10 diagnosis codes recorded in primary care, in- and out-patient care (information from EHIF treatment bills, E-Health epicrises, medical records from North Estonia Medical Centre and Tartu University Hospital), as well as the Causes of Death Register.

In MoBa, we used ICD-10 codes recorded in in-patient and outpatient care from the Norwegian Patient Registry comprising all registration in specialist health service in Norway from 2008 onwards^30^.

We studied clinical diagnosis of ADHD, MDD, anxiety disorder, stress-related disorder, bipolar disorder, and schizophrenia (eTable 3), as well as hyperlipidemia, obesity, T2D, hypertensive diseases, arteriosclerosis, ischemic heart diseases, heart failure, arrhythmias, thromboembolic disease, and cerebrovascular diseases (eTable 4). We did not consider clinical diagnosis of ADHD in STR due to low prevalence (Table 1).

In the STR, lifetime MDD was additionally assessed using the Composite International Diagnostic Interview Diagnostic Interview Short Form (CIDI-SF). Assessments in the SALT and STAGE cohorts were based on interviews by trained interviewers, and a combination of self-report and interviewers by trained interviewers, respectively (mean age at interview and standard deviation: 53±16).

### Statistical Modeling

We conducted logistic regression analysis to estimate odds ratios (ORs) for the associations between PRSs and 1) clinically diagnosed psychiatric disorders, and 2) clinically diagnosed CMDs. Associations were adjusted for ten genetic principal components (PCs), sex, and birth-year (modelled using a three degree-of-freedom natural spline term). Adjusted ORs (AORs) additionally controlled for all six PRSs, to adjust for pleiotropic effects across the six psychiatric disorders. In the STR, we further adjusted for correlation due to twinship using a sandwich estimator combined with generalized estimating equations (GEE). Pooled results were estimated using fixed effects meta-analysis. In a supplementary analysis, we additionally adjusted for self-reported BMI, assessed in the baseline surveys.

In the analysis of lifetime MDD assess using the CIDI-SF in the STR, we considered three different models. We first fitted logistic regression adjusting for age at interview using a three degree-of-freedom natural spline term, sex, as well as correlation due to twinship using a sandwich estimator combined with GEE. To conduct discordant twin-pair analysis, we used logistic regression conditioned on twin-pair identifier and include sex as the only covariate. We combined monozygotic and dizygotic twins in the analysis. For the analysis of specific CMDs, we excluded individuals with a previous diagnosis of the CMD prior to the interview date. Finally, we fitted conditional models additionally adjusting for self-reported BMI.

## Results

### Association of psychiatric PRSs with psychiatric diagnoses

**Figure 1.**
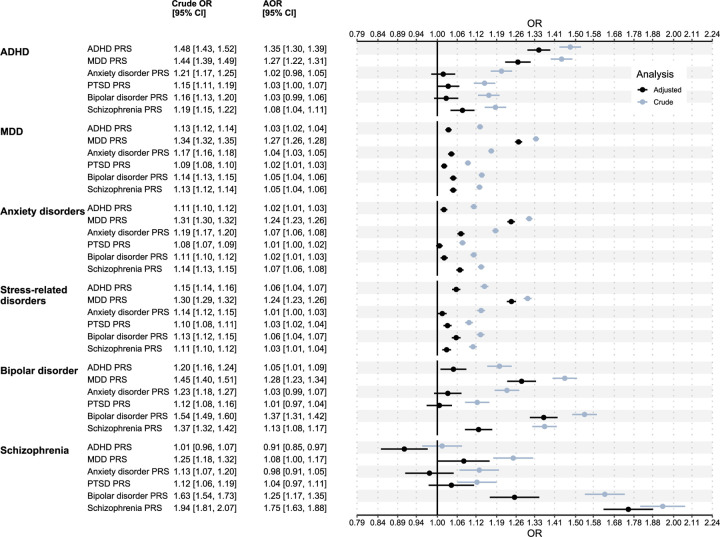
Associations of psychiatric PRSs with clinical diagnoses of psychiatric disorders. ORs were computed using logistic regression models with the PRS as the exposure variable and any recorded clinical diagnosis of the psychiatric disorder as the as the outcome variable. The crude model adjusted for principal components, sex, and birth-year. The adjusted model additionally adjusted for all psychiatric PRSs. ORs were computed by meta-analyzing ORs in STR, EstBB, and MoBa using fixed effects meta-analysis model. ADHD, attention deficit/hyperactivity disorder; AOR, adjusted odds ratio; MDD, major depressive disorder; OR, odds ratio; PTSD, post-traumatic stress disorder; PRS, polygenic risk score.

In the crude analysis, all PRSs showed positive associations with all clinically diagnosed psychiatric disorders (Figure 1), except that we did not observe an association between the ADHD PRS and clinically diagnosed schizophrenia. Cross-disorder associations were attenuated in the adjusted analysis indicating pleiotropic effects (Figure 1). ADHD, MDD, Bipolar disorder, and schizophrenia PRSs showed independent associations with clinical diagnoses of respective disorder. The strongest associations were found for the schizophrenia PRS (AOR and 95% CI per SD increase in PRS: 1.75 [1.63, 1.88]), the bipolar disorder PRS (AOR and 95% CI: 1.37 [1.31, 1.42]) and the ADHD PRS (AOR and 95% CI: 1.35 [1.3, 1.39]), for clinical diagnosis of each respective disorder. In contrast, the anxiety disorder and PTSD PRSs did not show strong associations with respective clinical diagnosis. Results from individual cohorts were similar, but EstBB showed consistently weaker effect sizes compared to STR and MoBa (eFigure 1)

### Association of psychiatric PRSs with CMD diagnoses

**Figure 2.**
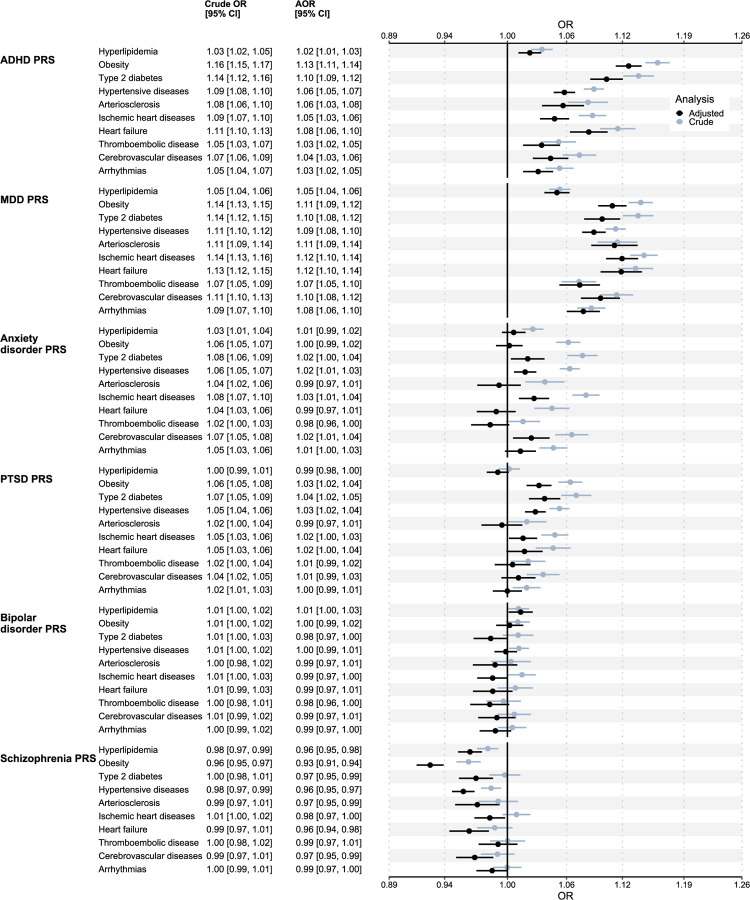
Associations of psychiatric PRSs with clinically diagnosed CMDs. ORs were computed using logistic regression models with the PRS as the exposure variable and any recorded clinical diagnosis of the CMD as the outcome variable. The crude model adjusted for principal components, sex, and birth-year. The adjusted model additionally adjusted for all psychiatric PRSs. ORs were computed by meta-analyzing ORs in STR, EstBB, and MoBa using a fixed effects meta-analysis model. ADHD, attention deficit/hyperactivity disorder; AOR, adjusted odds ratio; CMD, cardiometabolic disease; MDD, major depressive disorder; OR, odds ratio; PTSD, post-traumatic stress disorder; PRS, polygenic risk score.

Increased levels of the ADHD PRS were associated with increased risk of all CMDs in the crude analysis (Figure 2). AORs were attenuated but remained statistically significant. The ADHD PRS showed the strongest associations with clinically diagnosed obesity and T2D out of all psychiatric PRSs (AOR and 95% CI per SD increase in PRS: 1.13 [1.11, 1.14] and 1.10 [1.09, 1.12] respectively; Figure 2). Individual cohorts showed similar results, although associations were consistently weaker in the EstBB cohort (eFigure 2). Associations of the PRS based on genome-wide significant variants were attenuated, although statistically significant associations were still noted for obesity, T2D, heart failure, and thromboembolic disease (eFigures 3–4). When adjusting for BMI levels, AORs for clinically diagnosed obesity, T2D, and hypertensive diseases were attenuated (CIs were not overlapping; eFigure 5) but remained statistically significant.

Increased levels of MDD PRS were associated with increased risk of all CMDs across analyses and cohorts. The MDD PRS showed consistently stronger associations with CVDs compared to the other psychiatric PRSs (the difference was statistically significant for 5 of the 7 CVDs in the adjusted analysis; Figure 2). The strongest associations were found between the MDD PRS and ischemic heart disease (AOR and 95% CI per SD increase in PRS: 1.12 [1.10, 1.14]; Figure 2). Results were similar between the individual cohorts. The MDD PRS based on genome-wide significant variants showed statistically significant associations with all CMDs, except thromboembolic disease, although the ORs were attenuated (eFigures 3–4). AORs were not substantially affected by adjusting for BMI levels (eFigure 5).

In contrast to other psychiatric PRSs, increased levels of schizophrenia PRSs were associated with a decreased risk of all CMDs, except thromboembolic disease (Figure 2). The adjusted analysis showed stronger inverse associations compared to the crude analysis. The strongest inverse association was noted for clinically diagnosed obesity (OR and 95% CI per SD increase in PRS: 0.93 [0.91, 0.94]). When adjusting for BMI levels, AORs for obesity and hypertensive diseases were attenuated.

PRSs for anxiety disorder and PTSD showed weaker positive associations with CMDs compared to PRSs of MDD and ADHD (Figure 2). The associations were substantially attenuated in the adjusted analysis compared to the crude analysis (Figure 2; eFigure 2). The greatest AOR was noted for the association of PTSD PRS with T2D (AOR and 95% CI per SD increase in PRS: 1.04 [1.02, 1.05]; Figure 2). Finally, the PRS of bipolar disorder was not associated with increased risk of any CMD (Figure 2).

### Self-reported MDD and CMD diagnoses

**Figure 3.**
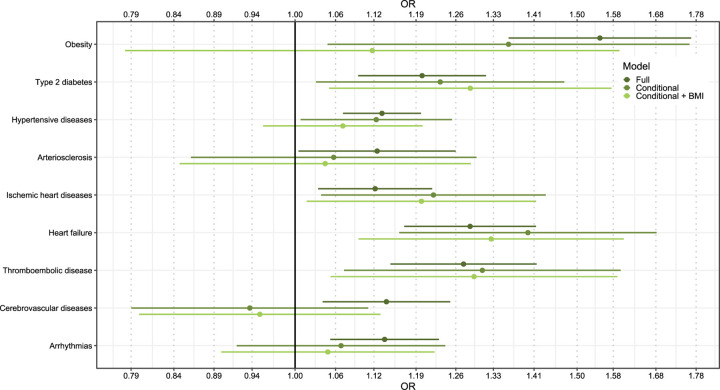
Association of self-reported lifetime MDD with CMD. **“**Full” indicates that estimates are obtained from analysis not conditioned on twin-pair, “Conditional” indicates that estimates are obtained by comparing twins that endorsed lifetime MDD in the CIDI-SF with their unexposed co-twins (i.e., after adjustment for factors shared between co-twins), and “Conditional + BMI” indicates that estimates are obtained in a conditional analysis that have also been adjusted for self-reported BMI levels reported in the same interview that assessed MDD. BMI, body mass index; CMD, cardiometabolic disease; MDD, major depressive disorder; CIDI-SF, Composite International Diagnostic Interview Short Form.

In the unconditional analysis, we found that CIDI-SF-assessed MDD was associated with increased risk of all CMDs, except arteriosclerosis (Figure 3). The associations remained statistically significant when comparing discordant co-twins, except for cerebrovascular diseases and arrhythmias. We found that a twin endorsing MDD in the CIDI-SF had on average a 40%, 31%, 23%, and 22% increased risk of being diagnosed with heart failure, thromboembolic disease, T2D, and ischemic heart diseases later in life, compared to their unexposed co-twin (*P* < 0.016). ORs were not substantially affected by adjusting for BMI, except for clinically diagnosed obesity.

## Discussion

Higher levels of ADHD and MDD PRSs were robustly associated with an increased risk of CMDs, whereas PRSs for other psychiatric disorders showed no such associations. Notably, the ADHD PRS was more strongly linked to metabolic diseases, while the MDD PRS was more strongly linked to CVDs. Moreover, discordant twin analyses demonstrated that self-reported MDD was associated with an increased risk of subsequent clinical diagnosis of CVD—even after adjusting for familial factors shared between co-twins. In contrast, higher schizophrenia PRSs were associated with a decreased risk of all CMDs (except for thromboembolic disease).

In the crude analysis, psychiatric PRSs were associated with clinical diagnoses of all psychiatric disorders. However, once we mutually adjusted for psychiatric PRSs, many cross-disorder associations were substantially attenuated, highlighting the large degree of pleiotropy between psychiatric disorders, and emphasizing the importance of mutually adjusting for psychiatric PRSs. In the adjusted analysis, ADHD, MDD, bipolar disorder and schizophrenia PRSs showed robust associations with their respective clinical diagnoses. Anxiety disorders and PTSD PRSs exhibited weaker associations with clinical diagnoses. Given their lower heritability and the smaller sample sizes of the discovery GWAS, the PRS predictions for these conditions might be less precise than for the other psychiatric disorders. Furthermore, the controls for the PTSD GWAS were trauma-exposed^25^, and therefore the results might not generalize to our population-based cohorts.

Higher levels of ADHD PRSs were robustly associated with an increased risk of all evaluated CMDs. In particular, the ADHD PRS showed stronger associations with clinically diagnosed obesity and T2D compared to other psychiatric PRSs. Notably, the AORs showed similar patterns to ORs estimated in a Swedish nationwide EHR-based sibling-controlled analysis^31^, providing a complementary line of evidence for the relationship between ADHD and CMD. Furthermore, the ADHD PRS derived from genome-wide significant variants also showed associations with increased risk of obesity and T2D, a result that should be less affected by horizontal pleiotropy compared to associations of the PRS based on all genetic variants.

Increased levels of MDD PRSs were also robustly associated with increased risk of all evaluated CMDs. Notably, the MDD PRS showed distinctive patterns of increased CVD risk relative to other psychiatric PRSs. Associations persisted for the PRS based on genome-wide significant variants, suggesting that associations are not fully explained by horizontal pleiotropy. Moreover, twins endorsing lifetime MDD in the CIDI-SF had greater risk of being diagnosed with CMDs compared to their unexposed co-twin. The PRS analysis evaluates genetic overlap between MDD and CMDs, while the discordant twin analysis evaluates the association of MDD with CMD adjusted on familial factors shared between co-twins. These analyses therefore provide complementary insights into the relationship between MDD and CMD. Combined with recent results based on time-to-event analysis in nationwide EHRs^5^ and two-sample Mendelian randomization^32^, our results offer increasingly robust support for the association between MDD and elevated CMD risk.

Conversely, higher schizophrenia PRSs were associated with a decreased risk of all CMDs (except for thromboembolic disease), in contrast with some previous reports^9,10,33^ but consistent with studies demonstrating a negative genetic correlation between schizophrenia and cardiometabolic risk factors^34–36^. These inverse associations became more pronounced after adjusting for other psychiatric PRSs, suggesting that pleiotropy with MDD and ADHD might have inflated the crude estimates. Individuals without a diagnosis of schizophrenia can still exhibit high schizophrenia PRSs^8^. Therefore, the observed association between higher schizophrenia PRSs and reduced cardiometabolic risk in our population-based cohorts might be driven by individuals without diagnosed schizophrenia. This implies that the well-documented phenotypic association between schizophrenia and increased cardiometabolic risk may not be related to genetic factors but rather to the consequences of treatments or disease manifestations that are absent in subclinical and untreated cases.

ADHD and MDD are associated with BMI^37,38^, which could explain the observed associations of ADHD and MDD PRSs with CMD risk. When adjusting for BMI, the associations for ADHD PRSs were attenuated but remained statistically significant for all CMDs, except for hyperlipidemia and thromboembolic disease. Notably, for clinically diagnosed obesity, T2D, and hypertensive diseases, AOR attenuation was statistically significant, indicating that BMI plays a role. In contrast, BMI adjustment had little impact on the investigated relationships between MDD and CMDs, suggesting that BMI does not mediate the link between MDD susceptibility and CMD risk. Interestingly, including BMI as a covariate weakened the protective associations observed with schizophrenia PRSs for clinically diagnosed obesity and hypertensive diseases, suggesting that higher schizophrenia PRSs may be associated with lower BMI levels, which in turn decreases the risk of CMDs, in line with previous reports^35^. However, results from the BMI adjusted PRS analysis might be affected by bias due to collider paths and need to be further corroborated with careful mediation analysis.

### Limitations

Interpreting associations between psychiatric PRSs and CMDs remains challenging, as they may reflect not only a direct effect of genetic risk but also consequences of treatment, subclinical symptoms, or other underlying pathophysiological mechanisms and pleiotropic effects. Moreover, psychiatric comorbidities were not excluded from the GWAS samples, potentially exacerbating cross-disorder pleiotropy. Nonetheless, our consistent findings across crude analyses, mutually adjusted models, and analyses based solely on genome-wide significant variants suggest that horizontal pleiotropy does not fully account for the observed associations. For MDD, the discordant twin analysis further strengthens evidence for a direct relationship with CMD risk. Another limitation is that all PRSs were derived from European-ancestry GWASs and applied to three Northern European cohorts, which may limit the generalizability of our findings to other populations. Finally, differences in heritability, polygenicity, and GWAS sample sizes across psychiatric disorders—particularly for anxiety disorder and PTSD—complicate comparisons of association strengths.

## Conclusions

Our analyses across three large, population-based cohorts provide robust evidence that elevated PRSs for ADHD and MDD are associated with an increased risk of CMDs. The association of self-reported MDD and subsequent CMD diagnosis further increase the clinical relevance of the findings. Collectively, these results suggest that primary prevention and improved treatment strategies may reduce CMD risk in individuals predisposed to MDD. Similarly, the observed link between polygenic risk for ADHD and increased risk of obesity and T2D supports the implementation of preventive lifestyle interventions aimed at mitigating metabolic disease risk in patients with ADHD. In contrast, the inverse association between schizophrenia PRSs and CMDs indicates that the well-documented phenotypic link between schizophrenia and CMDs may be driven by treatment effects or disease-specific manifestations rather than shared genetic factors.

## Supplementary Material

Supplement 1

## Figures and Tables

**Table 1.** Description of the STR, EstBB, and MoBa cohorts.

		STR genotyped	STR	EstBB	MoBa
**Sample size**		17,378	70,619	208,383	130,063
**Attained age, median (IQR), y**		71 (65–77)	70 (59–81)	50 (38–64)	48 (41–55)
**Age at interview, median (IQR), y**		56 (45–67)	51 (28–74)		
**Registers assessed**		In-patient, Out-patient, Cause-of-death	In-patient, Out-patient, Cause-of-death	Primary care, In-patient, Out-patient, Cause-of-death	In-patient, outpatient
**Start of follow-up, y**		1969	1969	2004	2008
**Start of follow-up with full coverage, y**		In-patient (1987), Outpatient (2001), Cause-of-death (1952)	In-patient (1987), Outpatient (2001), Cause-of-death (1973)	2004	2008
**End of follow-up**		2016-12-31	2016-12-31	2022-12-31; 2023-03 (Cause-of-death)	2022
**Sex, n (%)**	**Female**	9,046 (52.1)	38,288 (54.2)	136,319 (65.4)	77,122 (59.3)
	**Male**	8,332 (47.9)	32,331 (45.8)	72,064 (34.6)	52,941 (40.7)
**Psychiatric disorders, n (%)**	**ADHD**	11 (0.1)	298 (0.4)	1,676 (0.8)	2,360 (1.8)
	**MDD**	862 (5.0)	4,436 (6.3)	56,749 (27.2)	11,054 (8.5)
	**Anxiety disorders**	505 (2.9)	2,957 (4.2)	46,722 (22.4)	8,337 (6.4)
	**Stress-related disorders**	350 (2.0)	1,989 (2.8)	23,159 (11.1)	10,027 (7.7)
	**Bipolar disorder**	147 (0.80)	747 (1.1)	1,497 (0.7)	1,422 (1.1)
	**Schizophrenia**	26 (0.1)	217 (0.3)	1,057 (0.5)	73 (0.1)
**Metabolic disorders, n (%)**	**Hyperlipidemia**	0 (0)	0 (0)	62,553 (30.0)	2,268 (1.7)
	**Type 2 diabetes**	1,568 (9.0)	5,644 (8.0)	16,592 (8.0)	2,531 (1.9)
	**Obesity**	390 (2.2)	1,636 (2.3)	33,767 (16.2)	
**CVDs, n (%)**	**Ischemic heart disease**	2,537 (14.6)	9,426 (13.3)	28,213 (13.5)	2,249 (1.7)
	**Heart failure**	1,240 (7.1)	6,236 (8.8)	18,871 (9.1)	541 (0.4)
	**Cerebrovascular disease**	1,547 (8.9)	6,684 (9.5)	18,042 (8.7)	
	**Thromboembolic disease**	967 (5.6)	3,397 (4.8)	14,267 (6.8)	
	**Arrhythmias**	2,351 (13.5)	8,604 (12.2)	24,647 (11.8)	
	**Arteriosclerosis**	822 (4.7)	3,493 (4.9)	11,686 (5.6)	
	**Hypertensive diseases**	5,437 (31.3)	16,454 (23.3)	75,667 (36.3)	5,976 (4.6)

ADHD, attention deficit/hyperactivity disorder; AOR, adjusted odds ratio; EstBB, Estonian Biobank; IQR, interquartile range; MDD, major depressive disorder; PRS, polygenic risk score; STR, Swedish Twin Registry.

## References

[1] Liu NH, Daumit GL, Dua T, Excess mortality in persons with severe mental disorders: a multilevel intervention framework and priorities for clinical practice, policy and research agendas. World Psychiatry. Feb 2017;16(1):30–40. doi:10.1002/wps.20384 28127922 PMC5269481

[2] Plana-Ripoll O, Pedersen CB, Agerbo E, A comprehensive analysis of mortality-related health metrics associated with mental disorders: a nationwide, register-based cohort study. Lancet. Nov 16 2019;394(10211):1827–1835. doi:10.1016/S0140-6736(19)32316-5 31668728

[3] Tesli M, Degerud E, Plana-Ripoll O, Educational attainment and mortality in schizophrenia. Acta Psychiatr Scand. May 2022;145(5):481–493. doi:10.1111/acps.13407 35152418 PMC9305099

[4] Meijsen J, Hu K, Krebs MD, Quantifying the relative importance of genetics and environment on the comorbidity between mental and cardiometabolic disorders using 17 million Scandinavians. Nat Commun. Jun 13 2024;15(1):5064. doi:10.1038/s41467-024-49507-3 38871766 PMC11176385

[5] Shen Q, Mikkelsen DH, Luitva LB, Psychiatric disorders and subsequent risk of cardiovascular disease: a longitudinal matched cohort study across three countries. EClinicalMedicine. Jul 2023;61:102063. doi:10.1016/j.eclinm.2023.102063 37425374 PMC10329128

[6] Adams MJ, Streit F, Meng X, Trans-ancestry genome-wide study of depression identifies 697 associations implicating cell types and pharmacotherapies. Cell. 2025;doi:10.1016/j.cell.2024.12.002 PMC11829167 39814019

[7] Trubetskoy V, Pardinas AF, Qi T, Mapping genomic loci implicates genes and synaptic biology in schizophrenia. Nature. Apr 2022;604(7906):502–508. doi:10.1038/s41586-022-04434-5 35396580 PMC9392466

[8] Sullivan PF, Geschwind DH. Defining the Genetic, Genomic, Cellular, and Diagnostic Architectures of Psychiatric Disorders. Cell. Mar 21 2019;177(1):162–183. doi:10.1016/j.cell.2019.01.015 30901538 PMC6432948

[9] Bigdeli TB, Voloudakis G, Barr PB, Penetrance and Pleiotropy of Polygenic Risk Scores for Schizophrenia, Bipolar Disorder, and Depression Among Adults in the US Veterans Affairs Health Care System. JAMA Psychiatry. Sep 14 2022;79(11):1092–101. doi:10.1001/jamapsychiatry.2022.2742 36103194 PMC9475441

[10] Veeneman RR, Vermeulen JM, Bialas M, Mental illness and cardiovascular health: observational and polygenic score analyses in a population-based cohort study. Psychol Med. Apr 2024;54(5):931–939. doi:10.1017/S0033291723002635 37706306

[11] Haan E, Krebs K, Vosa U, Associations between attention-deficit hyperactivity disorder genetic liability and ICD-10 medical conditions in adults: utilizing electronic health records in a Phenome-Wide Association Study. Psychol Med. Jul 2024;54(10):2468–2481. doi:10.1017/S0033291724000606 38563284

[12] Du Rietz E, Xie T, Wang R, The contribution of attention-deficit/hyperactivity disorder polygenic load to metabolic and cardiovascular health outcomes: a large-scale population and sibling study. Transl Psychiatry. Nov 13 2024;14(1):470. doi:10.1038/s41398-024-03178-2 39537628 PMC11561358

[13] Garcia-Argibay M, du Rietz E, Lu Y, The role of ADHD genetic risk in mid-to-late life somatic health conditions. Transl Psychiatry. Apr 11 2022;12(1):152. doi:10.1038/s41398-022-01919-9 35399118 PMC8995388

[14] Pathak GA, Singh K, Choi KW, Genetic Liability to Posttraumatic Stress Disorder Symptoms and Its Association With Cardiometabolic and Respiratory Outcomes. JAMA Psychiatry. Jan 1 2024;81(1):34–44. doi:10.1001/jamapsychiatry.2023.4127 37910111 PMC10620678

[15] Grotzinger AD, Mallard TT, Akingbuwa WA, Genetic architecture of 11 major psychiatric disorders at biobehavioral, functional genomic and molecular genetic levels of analysis. Nat Genet. May 2022;54(5):548–559. doi:10.1038/s41588-022-01057-4 35513722 PMC9117465

[16] Zagai U, Lichtenstein P, Pedersen NL, Magnusson PKE. The Swedish Twin Registry: Content and Management as a Research Infrastructure. Twin Res Hum Genet. Dec 2019;22(6):672–680. doi:10.1017/thg.2019.99 31747977

[17] Lichtenstein P, Sullivan PF, Cnattingius S, The Swedish Twin Registry in the third millennium: an update. Twin Res Hum Genet. Dec 2006;9(6):875–82. doi:10.1375/183242706779462444 17254424

[18] Leitsalu L, Haller T, Esko T, Cohort Profile: Estonian Biobank of the Estonian Genome Center, University of Tartu. Int J Epidemiol. Aug 2015;44(4):1137–47. doi:10.1093/ije/dyt268 24518929

[19] Ojalo T, Haan E, Koiv K, Cohort Profile Update: Mental Health Online Survey in the Estonian Biobank (EstBB MHoS). Int J Epidemiol. Feb 14 2024;53(2)doi:10.1093/ije/dyae017 PMC10881104 38381979

[20] Milani L, Alver M, Laur S, From Biobanking to Personalized Medicine: the journey of the Estonian Biobank. medRxiv. 2024:2024.09.22.24313964. doi:10.1101/2024.09.22.24313964

[21] Magnus P, Birke C, Vejrup K, Cohort Profile Update: The Norwegian Mother and Child Cohort Study (MoBa). Int J Epidemiol. Apr 2016;45(2):382–8. doi:10.1093/ije/dyw029 27063603

[22] Demontis D, Walters GB, Athanasiadis G, Genome-wide analyses of ADHD identify 27 risk loci, refine the genetic architecture and implicate several cognitive domains. Nat Genet. Feb 2023;55(2):198–208. doi:10.1038/s41588-022-01285-8 36702997 PMC10914347

[23] Als TD, Kurki MI, Grove J, Depression pathophysiology, risk prediction of recurrence and comorbid psychiatric disorders using genome-wide analyses. Nat Med. Jul 2023;29(7):1832–1844. doi:10.1038/s41591-023-02352-1 37464041 PMC10839245

[24] Purves KL, Coleman JRI, Meier SM, A major role for common genetic variation in anxiety disorders. Mol Psychiatry. Dec 2020;25(12):3292–3303. doi:10.1038/s41380-019-0559-1 31748690 PMC7237282

[25] Nievergelt CM, Maihofer AX, Klengel T, International meta-analysis of PTSD genome-wide association studies identifies sex- and ancestry-specific genetic risk loci. Nat Commun. Oct 8 2019;10(1):4558. doi:10.1038/s41467-019-12576-w 31594949 PMC6783435

[26] Mullins N, Forstner AJ, O’Connell KS, Genome-wide association study of more than 40,000 bipolar disorder cases provides new insights into the underlying biology. Nat Genet. Jun 2021;53(6):817–829. doi:10.1038/s41588-021-00857-4 34002096 PMC8192451

[27] Schizophrenia Working Group of the Psychiatric Genomics C. Biological insights from 108 schizophrenia-associated genetic loci. Nature. Jul 24 2014;511(7510):421–7. doi:10.1038/nature13595 25056061 PMC4112379

[28] Lloyd-Jones LR, Zeng J, Sidorenko J, Improved polygenic prediction by Bayesian multiple regression on summary statistics. Nat Commun. Nov 8 2019;10(1):5086. doi:10.1038/s41467-019-12653-0 31704910 PMC6841727

[29] Choi SW, O’Reilly PF. PRSice-2: Polygenic Risk Score software for biobank-scale data. Gigascience. Jul 1 2019;8(7)doi:10.1093/gigascience/giz082 PMC6629542 31307061

[30] Bakken IJ, Ariansen AMS, Knudsen GP, Johansen KI, Vollset SE. The Norwegian Patient Registry and the Norwegian Registry for Primary Health Care: Research potential of two nationwide health-care registries. Scand J Public Health. Feb 2020;48(1):49–55. doi:10.1177/1403494819859737 31288711

[31] Du Rietz E, Brikell I, Butwicka A, Mapping phenotypic and aetiological associations between ADHD and physical conditions in adulthood in Sweden: a genetically informed register study. Lancet Psychiatry. Sep 2021;8(9):774–783. doi:10.1016/S2215-0366(21)00171-1 34242595 PMC8376653

[32] Bergstedt J, Pasman JA, Ma Z, Distinct biological signature and modifiable risk factors underlie the comorbidity between major depressive disorder and cardiovascular disease. Nat Cardiovasc Res. 2024;3(6):754–769. doi:10.1038/s44161-024-00488-y PMC11182748 39215135

[33] Veeneman RR, Vermeulen JM, Abdellaoui A, Exploring the Relationship Between Schizophrenia and Cardiovascular Disease: A Genetic Correlation and Multivariable Mendelian Randomization Study. Schizophr Bull. Mar 1 2022;48(2):463–473. doi:10.1093/schbul/sbab132 34730178 PMC8886584

[34] Bahrami S, Steen NE, Shadrin A, Shared Genetic Loci Between Body Mass Index and Major Psychiatric Disorders: A Genome-wide Association Study. JAMA Psychiatry. May 1 2020;77(5):503–512. doi:10.1001/jamapsychiatry.2019.4188 31913414 PMC6990967

[35] Reponen EJ, Ueland T, Rokicki J, Polygenic risk for schizophrenia and bipolar disorder in relation to cardiovascular biomarkers. Eur Arch Psychiatry Clin Neurosci. Aug 2024;274(5):1223–1230. doi:10.1007/s00406-023-01591-0 37145175 PMC11226473

[36] Rodevand L, Rahman Z, Hindley GFL, Characterizing the Shared Genetic Underpinnings of Schizophrenia and Cardiovascular Disease Risk Factors. Am J Psychiatry. Nov 1 2023;180(11):815–826. doi:10.1176/appi.ajp.20220660 37752828 PMC11780279

[37] Milaneschi Y, Simmons WK, van Rossum EFC, Penninx BW. Depression and obesity: evidence of shared biological mechanisms. Mol Psychiatry. Jan 2019;24(1):18–33. doi:10.1038/s41380-018-0017-5 29453413

[38] Cortese S, Moreira-Maia CR, St Fleur D, Morcillo-Penalver C, Rohde LA, Faraone SV. Association Between ADHD and Obesity: A Systematic Review and Meta-Analysis. Am J Psychiatry. Jan 2016;173(1):34–43. doi:10.1176/appi.ajp.2015.15020266 26315982

