# Associations of Genetic Liability to Six Psychiatric Disorders With Cardiometabolic Diseases

**DOI:** 10.1101/2025.03.11.25323757

**Published:** 2025-09-11

**Authors:** Jacob Bergstedt, Kadri Kõiv, Andreas Jangmo, Marit Haram, Piotr P. Jaholkowski, Jorien L. Treur, Isabell Brikell, Zheng Chang, Henrik Larsson, Patrik K. E. Magnusson, Andrew M. McIntosh, Cathryn M. Lewis, Brian K. Lee, Ida E. Sønderby, Yi Lu, Patrick F. Sullivan, Unnur A. Valdimarsdóttir, Ole Andreassen, Martin Tesli, Kelli Lehto, Fang Fang

**Affiliations:** 1 Unit of Integrative Epidemiology, Institute of Environmental Medicine, Karolinska Institutet, Stockholm, Sweden; 2 Estonian Genome Centre, Institute of Genomics, University of Tartu, Tartu, Estonia; 3 Department of Chronic Diseases, Norwegian Institute of Public Health, Oslo, Norway; 4 Department of Mental Health and Suicide, Norwegian Institute of Public Health, Oslo, Norway; 5 Division of Mental Health and Addiction, Oslo University Hospital, Oslo, Norway; 6 Institute of Clinical Medicine, University of Oslo, Oslo, Norway; 7 Center for Precision Psychiatry, University of Oslo, Oslo, Norway; 8 Genetic Epidemiology, Department of Psychiatry, Amsterdam UMC, University of Amsterdam, Amsterdam, Netherlands; 9 Department of Medical Epidemiology and Biostatistics, Karolinska Institutet, Stockholm, Sweden; 10 Department of Global Public Health and Primary Care, University of Bergen, Bergen, Norway; 11 Department of Biomedicine, Aarhus University, Aarhus, Denmark; 12 School of Medical Sciences, Faculty of Medicine and Health, Örebro University, Örebro, Sweden; 13 Centre for Clinical Brain Sciences, University of Edinburgh, Royal Edinburgh Hospital, Edinburgh, UK; 14 Centre for Genomics and Experimental Medicine, University of Edinburgh, Edinburgh, UK; 15 Social, Genetic and Developmental Psychiatry Centre, King’s College London, London, UK; 16 Department of Medical and Molecular Genetics, King’s College London, London, UK; 17 A.J. Drexel Autism Institute, Drexel University, Philadelphia, PA, USA; 18 Dornsife School of Public Health, Drexel University, Philadelphia, PA, USA; 19 K.G. Jebsen Centre for Neurodevelopmental Disorders, University of Oslo and Oslo University Hospital, Oslo, Norway; 20 Department of Medical Genetics, Oslo University Hospital and University of Oslo, Norway; 21 Department of Psychiatry, University of North Carolina at Chapel Hill, Chapel Hill, NC, USA; 22 Department of Genetics, University of North Carolina at Chapel Hill, Chapel Hill, NC, USA; 23 Centre of Public Health Sciences, Faculty of Medicine, School of Health Sciences, University of Iceland, Reykjavik, Iceland; 24 Department of Epidemiology, Harvard TH Chan School of Public Health, Harvard University, Boston, MA, USA; 25 Division of Mental Health and Substance Abuse, Diakonhjemmet Hospital, Oslo, Norway; 26 Department of Psychiatry, Østfold Hospital, Grålum, Norway

## Abstract

**IMPORTANCE:**

Individuals with psychiatric disorders have increased risk of cardiometabolic diseases (CMDs). Evaluating how psychiatric genetic liability relates to CMD may clarify mechanisms.

**OBJECTIVE:**

Identify genetic overlap between psychiatric disorders and CMDs independent of cross-disorder pleiotropy, BMI, and smoking.

**DESIGN, SETTING, AND PARTICIPANTS:**

Three Northern European cohorts (the Swedish Twin Registry, the Estonian Biobank, and the Norwegian Mother, Father and Child Cohort Study [MoBa]) totaling 355,159 individuals. Associations with CMDs were estimated as adjusted odds ratios (AORs) from logistic models mutually adjusted for all psychiatric PRSs and in models additionally adjusting for body mass index (BMI) and smoking. Cohort-specific AORs were pooled by inverse-variance weighting.

**MAIN OUTCOMES AND MEASURES:**

Exposures were PRSs for attention-deficit/hyperactivity disorder (ADHD), major depressive disorder (MDD), anxiety disorder, posttraumatic stress disorder (PTSD), bipolar disorder, and schizophrenia. Outcomes were diagnoses of CMDs (hyperlipidemia, obesity, type 2 diabetes, hypertensive diseases, arteriosclerosis, ischemic heart disease, heart failure, thromboembolic disease, cerebrovascular disease, and arrhythmias), ascertained from electronic health records.

**RESULTS:**

The MDD PRS was associated with increased risk of all CMDs across analyses (AORs ranged from 1.13 [95% CI, 1.10–1.15] for heart failure to 1.02 [95% CI, 1.00–1.05] for arrhythmias). The ADHD PRS was associated with increased risk of all CMDs (AOR ranged from 1.11 [95% CI, 1.09–1.12] for obesity to 1.02 [95% CI, 1.01–1.03] for hyperlipidemia), however associations where attenuated when adjusting for BMI and smoking (lifestyle adjusted AOR for obesity: 1.03 [95% CI, 1.02–1.05]). When not mutually adjusting for all psychiatric PRSs, anxiety disorder and PTSD PRSs were associated with all CMDs; these associations diminished after adjustment. The bipolar and schizophrenia PRSs were inversely associated with most CMDs (AOR for schizophrenia PRS and obesity, 0.93 [95% CI, 0.92–0.94]).

**CONCLUSIONS AND RELEVANCE:**

Associations between psychiatric PRSs and CMDs diverged: ADHD, MDD, anxiety disorder, and PTSD PRSs were positively associated with CMDs, whereas bipolar and schizophrenia PRSs were inversely associated. Genetic liability to MDD showed robust associations with CMDs independent of cross-disorder pleiotropy, BMI, and smoking status, whereas associations between the ADHD PRS and CMDs were largely attenuated after adjustment for BMI and smoking.

## Introduction

Cardiometabolic diseases (CMDs) reduce life expectancy in individuals with psychiatric disorders compared to the general population^1–3^. Studies using electronic health records have consistently shown that most psychiatric diagnoses are linked to CMD risk.^4,5^. Recent genome-wide association studies (GWASs) of psychiatric disorders have achieved sufficient statistical power to capture most common variant genetic risk^6,7^, which has enabled the construction of polygenic risk scores (PRSs; sums of risk alleles weighted by GWAS effect sizes) that capture individual-level genetic susceptibility^8^. Because PRSs are determined at birth, their associations with CMDs are immune to reverse causation and less prone to confounding than associations based on clinical diagnoses. PRS approaches have been employed to show that genetic liability to major depressive disorder (MDD)^9,10^, schizophrenia^9,10^, ADHD^11–13^, and post-traumatic stress disorder (PTSD)^14^ are associated with increased risk of CMDs. However, as psychiatric disorders exhibit substantial pleiotropy^15^, associations for a specific psychiatric PRS might reflect shared genetic background with other psychiatric disorders or lifestyle factors. Few studies have comprehensively mapped associations of genetic liability to psychiatric disorders with CMDs, while adjusting for other, genetically correlated, psychiatric PRSs or lifestyle factors.

We analyzed 355,159 genotyped participants from the population-based Swedish Twin Registry (STR), the volunteer-based Estonian Biobank (EstBB), and the population-based Norwegian Mother, Father, and Child Cohort Study (MoBa) to assess associations of PRSs for six psychiatric disorders (ADHD, MDD, anxiety disorder, PTSD, bipolar disorder, and schizophrenia) with clinical diagnoses of 10 CMDs. We estimated independent associations by mutually adjusting for all psychiatric PRSs and evaluated lifestyle influences through additional adjustment for body mass index (BMI) and smoking. Our primary objective was to identify genetic overlap between psychiatric disorders and CMDs independent of cross-disorder pleiotropy, BMI, and smoking, potentially reflecting shared pathophysiological pathways.

## Methods

### Study participants

#### Swedish Twin Registry

The Screening Across the Lifespan Twin (SALT) cohort of the STR is a population-based study of all Swedish twins born between 1911 and 1958 conducted 1998–2002 (response rate of 70%^16^). Two studies nested in SALT were later conducted with the aim of genotyping the individuals. In the TwinGene study conducted 2004–2008, 22,000 SALT participants born in 1943 or earlier were invited to donate blood (response rate of 56%)^17^. In the SALTY study conducted 2008–2010, 24,914 SALT participants born 1943–1958 were invited to provide a saliva sample (response rate of 47%^18^). After quality control preprocessing, the STR sample comprised 17,378 genotyped individuals (Table 1).

The use of the STR cohort in the present study was approved by the Swedish Ethical Review Authority (registration numbers 2021-03197, 2021-02994, 2021-02994).

#### Estonian Biobank

The EstBB is a volunteer-based biobank with a sample size of 210,000 participants, comprising around 20% of the adult population of Estonia^19–21^ linked to primary and specialist care records.

The activities of the EstBB are regulated by the Human Genes Research Act, which was adopted in 2000 specifically for the operations of the EstBB. Individual level data analysis in the EstBB was carried out under ethical approval no. 1.1-12/624 and its extensions from the Estonian Committee on Bioethics and Human Research (Estonian Ministry of Social Affairs), using data according to release application no. 6-7/GI/16153 from the Estonian Biobank.

#### MoBa

The MoBa study is conducted by the Norwegian Institute of Public Health. All pregnant women able to read Norwegian were eligible to participate in the study. Postal invitations were sent in advance of the first routine ultrasound examination in the 17^th^ week of pregnancy. Fathers were also invited to participate.

Participants were recruited 1999–2008 from all over Norway. The mothers consented to participation in 41% of the pregnancies. The cohort includes 114,500 individuals^22^. The establishment of MoBa and initial data collection was based on a license from the Norwegian Data Protection Agency and approval from The Regional committees for Medical and Health Research Ethics. The MoBa cohort is currently regulated by the Norwegian Health Registry Act. The use of MoBa cohort in the present study was approved by The Regional Committees for Medical and Health Research Ethics (REK 2016/1226).

### PRS profiling

We leveraged the most recent GWASs of ADHD^23^, MDD^6^, anxiety disorder^24^, PTSD^25^, bipolar disorder^26^, and schizophrenia^7^ to compute PRSs (eTable 1). Details on the genotyping in STR, EstBB, and MoBa are given in the Supplement (eMethods). If the discovery GWAS included samples from our study cohorts, we used leave-cohort-out GWAS summary statistics to compute PRSs. In EstBB, we removed 1,383 individuals (234 cases) that were included in a seminal schizophrenia GWAS study that formed the basis for subsequent studies^27^. We computed PRSs using the SBayesR framework^28^. We used a map of linkage disequilibrium estimated from 2,865,810 SNPs in 50,000 UK Biobank samples using a shrinkage estimator^28^. To reduce the contributions of horizontal pleiotropy, we additionally computed PRSs based solely on genome-wide significant variants using clumping and thresholding (P<5×10^−8^) implemented in PRSice-2^29^ (eTable 2). PRSs were standardized to have zero mean and a standard deviation of one.

### Outcome Measures

We ascertained psychiatric disorders and CMDs using electronic health records. We considered both main and secondary diagnoses. One diagnosis was sufficient to be identified as a case. To validate PRSs, we identified clinical diagnosis of ADHD, MDD, anxiety disorder, stress-related disorder, bipolar disorder, and schizophrenia (Table 1; eTable 3). Main outcomes were CMDs, including metabolic diseases (hyperlipidemia, type 2 diabetes [T2D], and obesity), hypertensive diseases, and cardiovascular diseases (CVDs; arteriosclerosis, ischemic heart diseases, heart failure, thromboembolic disease, cerebrovascular diseases, and arrhythmias; Table 1, eTable 4).

In STR, outcomes were identified from International Classification of Diseases (ICD)-8–10 codes in the Swedish National Patient Register (inpatient care nationwide since 1987 and specialist outpatient care with >80% national coverage since 2001) and from the Cause of Death Register, with follow-up through 2016. ADHD was excluded because of low prevalence (Table 1). Hyperlipidemia data were unavailable.

In the EstBB cohort, we used ICD-10 codes recorded in primary care, inpatient and outpatient care (Estonian Health Insurance Fund treatment bills, E-Health epicrises, and medical records from North Estonia Medical Centre and Tartu University Hospital), as well as the Cause of Death Register (since 2004), with follow-up through 2024.

In MoBa, we used ICD-10 codes from the Norwegian Patient Registry^30^ (all specialist health service visits nationwide since 2008) with follow-up through 2022. Data on obesity, cerebrovascular disease, thromboembolic disease, arrhythmias, and arteriosclerosis were not available for the MoBa cohort.

### Covariates

To assess the contribution of lifestyle factors, we adjusted for BMI and smoking. In the STR, BMI was computed from weight and height assessed in the SALT questionnaire (given 1998–2002). Smoking was also assessed in the SALT questionnaire and was categorized as: never (0), former (1), and current (2).

In the EstBB, BMI and smoking were assessed using the baseline questionnaire. BMI was computed from weight and height. Pregnant individuals at that time, extreme BMI outliers (values more than ±5 standard deviations from the mean), and BMI recorded before the age of 18 years were excluded. Smoking was categorized as: never (0), former (1), and current (2).

In the MoBa cohort, BMI and smoking status prior to pregnancy was assessed in baseline questionnaire that participating women answered at week 15 of pregnancy. The women reported on both their own and their partners BMI and smoking status. Since virtually no women smoked during pregnancy, smoking status in MoBa was assessed using the binary variable with categories: never (0), and former (1).

### Statistical Modeling

We conducted logistic regression analysis to estimate odds ratios (ORs) for the associations of psychiatric PRSs with psychiatric disorders (to validate PRSs) and CMDs. First, we performed a crude analysis where models were adjusted for the first ten genetic principal components (PCs), sex, and birth-year (modelled using a three degree-of-freedom natural spline term). To estimate adjusted ORs (AORs), we then conducted a mutually adjusted analysis additionally adjusted for all six PRSs, addressing pleiotropic effects across the six psychiatric disorders. In the STR cohort, we modelled sample correlation due to twinship using a sandwich estimator combined with generalized estimating equations (GEE). Pooled results were computed using inverse variance weighting of estimates from the three cohorts. Finally, we fitted models additionally adjusted for BMI and smoking status, to examine the potential influence of lifestyle factors on the studied associations. Proportion of variance explained on the liability scale, the logliability scale, and according to Nagelkerkes measure^31^ were estimated in models including the PRS as the only predictor. We consider an association statistically significant if *P*<0.05.

## Results

### Association of psychiatric PRSs with psychiatric diagnoses

Psychiatric PRSs showed liability-scale R^2^ explained in psychiatric clinical diagnoses consistent with the original GWAS publications (liability-scale R^2^ ranged from 1–10%; eTable 5). In the crude analysis, all PRSs showed positive associations with all psychiatric disorders (eFigure 1), except that the ADHD PRS showed no association with schizophrenia. Cross-disorder associations were attenuated in the analysis with mutual adjustment of all psychiatric PRSs, indicating contributions of pleiotropy (eFigure 1). The strongest associations with diagnosis of the respective disorders were found for the schizophrenia and bipolar disorder PRSs (AORs, 1.77 and 1.34; *P*<1.66×10^−47^; eFigure 1; eTable 6). Estimates from individual cohorts showed similar patterns (eFigure 2).

### Association of psychiatric PRSs with CMD diagnoses

**Figure 1.**
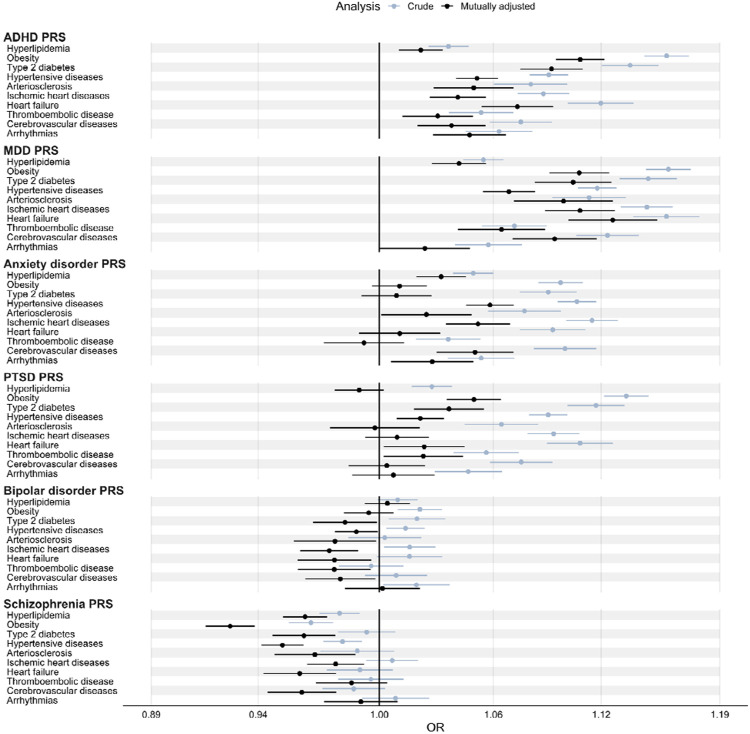
Associations of psychiatric PRSs with cardiometabolic diseases. In each cohort, ORs were computed from logistic regression models with the PRS as the exposure variable and any recorded clinical diagnosis of the cardiometabolic disease as the outcome variable. The crude model adjusted for the first 10 principal components, sex, and birth-year. The mutually adjusted model additionally adjusted for all other psychiatric PRSs. All ORs were computed from cohort-specific ORs using inverse variance weighting. All ORs correspond to the multiplicative change in odds per +1 standard deviation in PRS. ADHD, attention deficit/hyperactivity disorder; AOR, adjusted odds ratio; CMD, cardiometabolic disease; MDD, major depressive disorder; OR, odds ratio; PTSD, post-traumatic stress disorder; PRS, polygenic risk score.

In the crude analysis, ADHD, MDD, anxiety disorder, and PTSD PRSs were associated with increased risk of all CMDs. The strongest associations were found for the ADHD and MDD PRSs with obesity (OR=1.16 for both PRSs, *P*<3.31×10^−59^; Figure 1; eTable 7) and for the MDD PRSs with heart failure (OR=1.16; *P*=8.34×10^−65^; Figure 1; eTable 7). Individual cohorts showed similar results, although the associations were consistently weaker in the EstBB cohort (eFigure 3).

The ADHD PRS showed statistically significant associations with all CMDs in the analysis mutually adjusted for all PRSs. The strongest associations were found with obesity and T2D (AORs, 1.11 and 1.09; *P*<1.81×10^−27^; Figure 1; eTable 7). The ADHD PRS based on genome-wide significant variants showed statistically significant associations for obesity, T2D, heart failure, and thromboembolic disease (eFigures 4–5).

The MDD PRS was associated with increased risk of all CMDs (except thromboembolic disease) across analyses (i.e., in models mutually adjusted for all PRSs and for the PRS based on genome-wide significant variants). It showed stronger associations with CVDs compared to the other psychiatric PRSs (nonoverlapping CIs indicated higher AORs for arteriosclerosis, ischemic heart disease, and heart failure, compared to all other PRSs; Figure 1; eFigures 4–5; eTable 7). The strongest association was for heart failure (AOR, 1.13; *P*=9.17×10^−25^; Figure 1; eTable 7).

The anxiety disorder PRS was associated with hyperlipidemia, hypertensive diseases, ischemic heart diseases, and cerebrovascular disease across analyses (Figure1; eTable 7; eFigures 4–5). The strongest association was for hypertensive disease (AOR, 1.06; *P*=9.60×10^−20^; Figure1; eTable 7).

For the PTSD PRS, associations with hyperlipidemia, arteriosclerosis, ischemic heart diseases, cerebrovascular disease, and arrhythmias were no longer statistically significant in the mutually adjusted analysis. It was associated with obesity, T2D, and hypertensive disease across analyses. The strongest AOR was for found for obesity (AOR, 1.05; *P*=8.46×10^−12^; Figure 1; eFigures 4–5; eTable 7).

The bipolar disorder PRS showed weak but statistically significant associations with obesity, T2D, hypertensive disease, ischemic heart diseases, and arrhythmias in the crude analysis (ORs<1.02; *P*>3.34×10^−4^; Figure 1; eTable 7). In contrast, it showed inverse associations with all CMDs except hyperlipidemia and arrhythmias in the mutually adjusted analysis (Figure 1). The strongest inverse association was found for ischemic heart diseases (AOR=0.97; P=7.06×10^−4^; Figure 1; eTable 7).

In the crude analysis, the schizophrenia PRS showed statistically significant inverse associations with hyperlipidemia, obesity, and hypertensive diseases (strongest for obesity: OR=0.97; *P*=1.67×10^−9^; Figure 1; eTable 7). After mutually adjustment for all other psychiatric PRSs, it showed statistically significant inverse associations with all CMDs except for thromboembolic diseases, and arrhythmias (strongest for obesity: AOR=0.93; *P*=5.11×10^−33^; Figure 1; eTable 7).

### Associations after adjustment for smoking status and BMI

**Figure 2.**
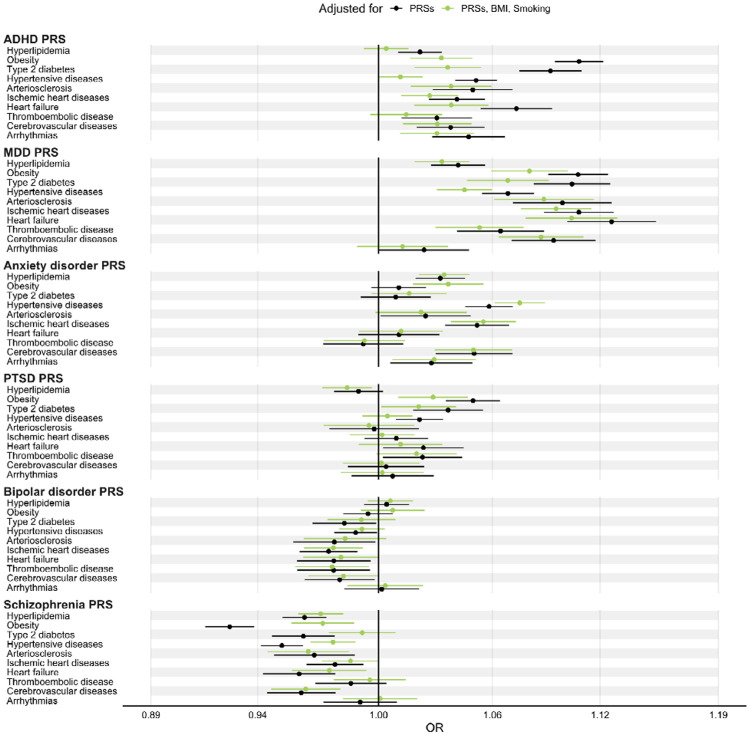
Associations of psychiatric PRSs with cardiometabolic diseases after adjustment for smoking status and BMI. In each cohort, ORs were computed from logistic regression models with the PRS as exposure variable and any recorded clinical diagnosis of the cardiometabolic disease as the outcome variable and all other psychiatric PRSs, sex, birth-year, and the 10 first genetic principal components as covariates. The “PRSs, BMI, Smoking” model additionally adjusted for BMI and smoking. All ORs were computed from cohort specific ORs by inverse variance weighting. All ORs correspond to the multiplicative change in odds per +1 standard deviation in PRS. ADHD, attention deficit/hyperactivity disorder; BMI, body mass index; CMD, cardiometabolic disease; MDD, major depressive disorder; OR, odds ratio; PTSD, post-traumatic stress disorder; PRS, polygenic risk score.

The ADHD PRS showed attenuated AORs with CMDs after adjustment for BMI and smoking (lifestyle adjusted AORs<1.04; *P*>4.6×10^−5^; Figure 2; eTable 8). The largest attenuations were found for hyperlipidemia, obesity, T2D, and hypertensive diseases (82%, 69%, 60%, and 78% attenuation respectively; Figure 2).

Associations for the MDD PRS were less affected than those for the ADHD PRS after adjustment for BMI and smoking. The largest attenuations were found for hypertensive diseases, T2D, and obesity (34%, 33%, and 24%). Cerebrovascular disease, arteriosclerosis, and ischemic heart disease showed limited attenuation (lifestyle adjusted AORs, 1.09, 1.09, and 1.10; reductions of 7.15%, 10.1%, and 11.4%; Figure 2; eTable 8). AORs for anxiety disorder, PTSD, and bipolar disorder PRSs showed also limited attenuation after adjustment for lifestyle factors.

In contrast, inverse associations of the schizophrenia PRS with obesity, T2D, and hypertensive diseases were attenuated after adjustment for BMI and smoking: ORs increased from 0.93 to 0.97, 0.96 to 0.99, and 0.95 to 0.98, respectively (reductions of 62.5%, 78.2%, and 52.8%).

## Discussion

The findings of this multinational study suggest that genetic liability to different psychiatric disorders show distinct patterns of association with CMDs. The MDD PRS showed the strongest associations with CMDs across analyses and cohorts. The ADHD PRS showed strong associations with obesity and T2D that were substantially attenuated when adjusting for BMI and smoking. In contrast, the schizophrenia PRS showed inverse associations with most CMDs across analyses and cohorts.

Psychiatric PRSs were positively associated with all psychiatric diagnoses in the crude analysis. After mutual adjustment for all psychiatric PRSs, most cross-disorder associations were substantially attenuated, consistent with extensive pleiotropy across disorders. Notably, the MDD PRS retained substantial cross-disorder associations even after mutual adjustment of all PRSs, suggesting that genetic overlap between other psychiatric PRSs and CMDs may be inflated by pleiotropy with MDD, highlighting the need for mutual adjustment.

The MDD PRS showed a distinctive association with increased CVD risk (arteriosclerosis, ischemic heart diseases, heart failure, thromboembolic disease, and cerebrovascular disease) relative to the other psychiatric PRSs. Combined with recent results based on time-to-event analysis in nationwide electronic health records^5^, genetic overlap estimated using GWAS summary data^32^, and two-sample Mendelian randomization^32^, our results add to increasingly robust support for a distinctive relationship between MDD and CVD.

The anxiety disorder PRS showed associations with increased risk of hyperlipidemia, hypertensive diseases, ischemic heart diseases, and cerebrovascular diseases across analyses providing robust evidence for a genetic link between anxiety disorder and CMDs, complementary to evidence based on registe-rbased analyses^5^.

The PTSD PRS was associated with an increased risk of all CMDs in the crude analysis, confirming previous studies^14^. However, in the mutually adjusted analysis, associations with arteriosclerosis, ischemic heart diseases, cerebrovascular diseases, and arrhythmias disappeared, suggesting that these associations might be related to cross-disorder pleiotropy.

Schizophrenia PRSs were associated with a decreased risk of most CMDs, in contrast with previous reports^9,10,33^, but consistent with studies demonstrating a negative genetic correlation between schizophrenia and cardiometabolic risk factors^34–36^. The inverse associations became more pronounced after adjusting for other psychiatric PRSs, suggesting that pleiotropy with other psychiatric disorders might have inflated the crude estimates. A previous report found that individuals with untreated schizophrenia showed consistently lower BMI trajectories between 20–40 years of age compared to controls, whereas individuals with treated schizophrenia showed markedly increased BMI trajectories compared to controls, suggesting that our results are more in line with previous observations for untreated schizophrenia than treated schizophrenia^37^.

Psychiatric disorders are associated with altered BMI^37–39^ and increased likelihood of smoking^40–42^, which could contribute to the observed associations of psychiatric PRSs with CMDs. The ADHD PRS showed strong associations with metabolic and hypertensive diseases; however, after adjustment for BMI and smoking, these associations were largely attenuated, indicating that BMI and smoking play an important role in these relationships. By contrast, adjustment for BMI and smoking had little impact on the associations of MDD, PTSD, and anxiety disorder PRSs with CVDs, suggesting that BMI and smoking do not explain these genetic links. Notably, including BMI and smoking status as covariates attenuated the inverse associations observed for schizophrenia PRSs, consistent with previous reports of inverse associations between schizophrenia PRSs and BMI^35^.

### Limitations

Interpretation of the associations between psychiatric PRSs and CMDs remains challenging, as they may reflect pleiotropic effects. Psychiatric comorbidities are rarely excluded from the existing GWAS samples, potentially exacerbating psychiatric cross-disorder pleiotropy. Yet, consistent findings across analyses suggest that horizontal pleiotropy does not fully account for our observed associations.

All PRSs were derived from European-ancestry GWASs and applied to three Northern European cohorts, which may limit generalizability to other populations. The cohorts also differed in health-care context and data sources: STR included inpatient and outpatient data, MoBa inpatient data only, and EstBB primary care plus inpatient and outpatient data. However, results were broadly similar across cohorts, suggesting robustness to these differences.

BMI and smoking were assessed at a single time point, which may not capture their full effects; future work should consider longitudinal measures. Moreover, conditioning on post-exposure BMI or smoking may induce collider bias by opening paths between the PRS and factors that confound BMI/smoking–outcome relationships. Because such bias would likely affect all estimates similarly, it is unlikely to explain differences between PRSs.

We cannot exclude survival bias as a contributor to the inverse associations for schizophrenia and bipolar PRSs. These disorders are associated with premature mortality^2^; high PRS individuals maybe die of other causes before developing CMDs. Yet most psychiatric disorders are linked to premature mortality^2^, and late-onset diseases such as ischemic heart disease, which should be most sensitive to survival bias, showed among the weakest inverse associations. Together, these results suggest survival bias is unlikely to fully account for the inverse associations of schizophrenia and bipolar PRSs with CMDs.

Finally, differences in heritability, polygenicity, and GWAS sample size across psychiatric disorders complicate direct comparisons of association magnitudes across PRSs.

## Conclusions

Our multinational analyses provide robust evidence that MDD genetic liability is associated with increased risk of CMDs independent of cross-disorder psychiatric pleiotropy, BMI, and smoking. Associations between ADHD PRSs and CMDs were largely attenuated after adjustment for BMI and smoking. In contrast, schizophrenia PRSs showed inverse associations with several CMDs, suggesting that the well-documented phenotypic association between schizophrenia and CMDs is unlikely to be explained by shared common genetic liability.

## Supplementary Material

Supplement 1

Supplement 2

## Figures and Tables

**Table 1.** Description of the Swedish Twin Registry, Estonian Biobank, and MoBa cohorts. Life-time diagnoses until the end of follow-up are described. Records for hyperlipidemia were not available for the STR cohort. Records of obesity, cerebrovascular disease, thromboembolic disease, arrhythmias, and arteriosclerosis were not available for the MoBa cohort. ADHD, attention deficit/hyperactivity disorder; EstBB, Estonian Biobank; IQR, interquartile range; MDD, major depressive disorder; STR, Swedish Twin Registry.

		STR	EstBB	MoBa
**Sample size**		17,378	208,384	129,955
**Age at end of follow-up, median (IQR), y**		71 (65–77)	51 (39–65)	48 (41–55)
**Registers assessed**		In/out-patient care, Cause-of-death	Primary care, In/out-patient care, Cause-of-death	In/out-patient care
**Start of follow-up, y**		1964	2004	2007
**Start of follow-up with full coverage, y**		In-patient care (1987), Out-patient care (2001), cause-of-death (2004)	2004	2007
**End of follow-up**		2016-12-31	2024-12-31	2022-12-31
**Sex, n (%)**	**Female**	9,046 (52.1)	136,318 (65.4)	77,064 (59.3)
	**Male**	8,332 (47.9)	72,066 (34.6)	52,891 (40.7)
**Psychiatric disorders, n (%)**	**ADHD**	11 (0.1)	3,282 (1.57)	2,360 (1.8)
	**MDD**	862 (5.0)	61,755 (29.6)	11,043 (8.5)
	**Anxiety disorders**	505 (2.9)	51,061 (24.5)	8,325 (6.4)
	**Stress-related disorders**	350 (2.0)	24,877 (11.9)	10,018 (7.7)
	**Bipolar disorder**	147 (0.80)	1,624 (0.8)	1,420 (1.1)
	**Schizophrenia**	26 (0.1)	1,084 (0.5)	73 (0.05)
**Metabolic disorders, n (%)**	**Hyperlipidemia**	NA	72,975 (35.0)	2,268 (1.7)
	**Type 2 diabetes**	1,568 (9.0)	18,528 (8.9)	2,526 (1.9)
	**Obesity**	390 (2.2)	38,355 (18.4)	NA
**Hypertensive diseases, n (%)**	**Hypertensive diseases**	5,437 (31.3)	81,040 (38.9)	5,971 (4.6)
**CVDs, n (%)**	**Ischemic heart disease**	2,537 (14.6)	31,899 (15.3)	2,248 (1.7)
	**Heart failure**	1,240 (7.1)	21,149 (10.2)	541 (0.4)
	**Cerebrovascular disease**	1,547 (8.9)	19,630 (9.4)	NA
	**Thromboembolic disease**	967 (5.6)	15,441 (7.4)	NA
	**Arrhythmias**	2,351 (13.5)	16,261 (7.8)	NA
	**Arteriosclerosis**	822 (4.7)	12,904 (6.2)	NA

## Key Points

### Question:

Is genetic liability to specific psychiatric disorders associated with an increased risk of clinically diagnosed cardiometabolic diseases even after adjusting for cross-disorder psychiatric pleiotropy, body mass index, and smoking status?

### Findings:

In this study of cohorts in Sweden, Estonia, and Norway, genetic liability to major depressive disorder (MDD) showed stronger associations with cardiometabolic disease (CMD) compared to other psychiatric disorders. Genetic liability to anxiety disorder and PTSD showed associations with CMD that were attenuated when adjusting for cross-disorder psychiatric pleiotropy. Genetic liability to ADHD showed strong associations with obesity and type 2 diabetes that were strongly attenuated when adjusting for body mass index, and smoking status. Genetic liability to schizophrenia showed inverse associations with CMD.

### Meaning:

These findings suggest that MDD has a distinctive genetic relationship with CMD compared to other psychiatric disorders and that the well-established phenotypic association between schizophrenia and CMD is not related to genetic factors.

## References

[1] Liu NH, Daumit GL, Dua T, Excess mortality in persons with severe mental disorders: a multilevel intervention framework and priorities for clinical practice, policy and research agendas. World Psychiatry. Feb 2017;16(1):30–40. doi:10.1002/wps.20384 28127922 PMC5269481

[2] Plana-Ripoll O, Pedersen CB, Agerbo E, A comprehensive analysis of mortality-related health metrics associated with mental disorders: a nationwide, register-based cohort study. Lancet. Nov 16 2019;394(10211):1827–1835. doi:10.1016/S0140-6736(19)32316-5 31668728

[3] Tesli M, Degerud E, Plana-Ripoll O, Educational attainment and mortality in schizophrenia. Acta Psychiatr Scand. May 2022;145(5):481–493. doi:10.1111/acps.13407 35152418 PMC9305099

[4] Meijsen J, Hu K, Krebs MD, Quantifying the relative importance of genetics and environment on the comorbidity between mental and cardiometabolic disorders using 17 million Scandinavians. Nat Commun. Jun 13 2024;15(1):5064. doi:10.1038/s41467-024-49507-3 38871766 PMC11176385

[5] Shen Q, Mikkelsen DH, Luitva LB, Psychiatric disorders and subsequent risk of cardiovascular disease: a longitudinal matched cohort study across three countries. EClinicalMedicine. Jul 2023;61:102063. doi:10.1016/j.eclinm.2023.102063 37425374 PMC10329128

[6] Adams MJ, Streit F, Meng X, Trans-ancestry genome-wide study of depression identifies 697 associations implicating cell types and pharmacotherapies. Cell. 2025;doi:10.1016/j.cell.2024.12.002 PMC11829167 39814019

[7] Trubetskoy V, Pardinas AF, Qi T, Mapping genomic loci implicates genes and synaptic biology in schizophrenia. Nature. Apr 2022;604(7906):502–508. doi:10.1038/s41586-022-04434-5 35396580 PMC9392466

[8] Sullivan PF, Geschwind DH. Defining the Genetic, Genomic, Cellular, and Diagnostic Architectures of Psychiatric Disorders. Cell. Mar 21 2019;177(1):162–183. doi:10.1016/j.cell.2019.01.015 30901538 PMC6432948

[9] Bigdeli TB, Voloudakis G, Barr PB, Penetrance and Pleiotropy of Polygenic Risk Scores for Schizophrenia, Bipolar Disorder, and Depression Among Adults in the US Veterans Affairs Health Care System. JAMA Psychiatry. Sep 14 2022;79(11):1092–101. doi:10.1001/jamapsychiatry.2022.2742 36103194 PMC9475441

[10] Veeneman RR, Vermeulen JM, Bialas M, Mental illness and cardiovascular health: observational and polygenic score analyses in a population-based cohort study. Psychol Med. Apr 2024;54(5):931–939. doi:10.1017/S0033291723002635 37706306

[11] Haan E, Krebs K, Vosa U, Associations between attention-deficit hyperactivity disorder genetic liability and ICD-10 medical conditions in adults: utilizing electronic health records in a Phenome-Wide Association Study. Psychol Med. Jul 2024;54(10):2468–2481. doi:10.1017/S0033291724000606 38563284

[12] Du Rietz E, Xie T, Wang R, The contribution of attention-deficit/hyperactivity disorder polygenic load to metabolic and cardiovascular health outcomes: a large-scale population and sibling study. Transl Psychiatry. Nov 13 2024;14(1):470. doi:10.1038/s41398-024-03178-2 39537628 PMC11561358

[13] Garcia-Argibay M, du Rietz E, Lu Y, The role of ADHD genetic risk in mid-to-late life somatic health conditions. Transl Psychiatry. Apr 11 2022;12(1):152. doi:10.1038/s41398-022-01919-9 35399118 PMC8995388

[14] Pathak GA, Singh K, Choi KW, Genetic Liability to Posttraumatic Stress Disorder Symptoms and Its Association With Cardiometabolic and Respiratory Outcomes. JAMA Psychiatry. Jan 1 2024;81(1):34–44. doi:10.1001/jamapsychiatry.2023.4127 37910111 PMC10620678

[15] Grotzinger AD, Mallard TT, Akingbuwa WA, Genetic architecture of 11 major psychiatric disorders at biobehavioral, functional genomic and molecular genetic levels of analysis. Nat Genet. May 2022;54(5):548–559. doi:10.1038/s41588-022-01057-4 35513722 PMC9117465

[16] Lichtenstein P, Sullivan PF, Cnattingius S, The Swedish Twin Registry in the third millennium: an update. Twin Res Hum Genet. Dec 2006;9(6):875–82. doi:10.1375/183242706779462444 17254424

[17] Magnusson PK, Almqvist C, Rahman I, The Swedish Twin Registry: establishment of a biobank and other recent developments. Twin Res Hum Genet. Feb 2013;16(1):317–29. doi:10.1017/thg.2012.104 23137839

[18] Zagai U, Lichtenstein P, Pedersen NL, Magnusson PKE. The Swedish Twin Registry: Content and Management as a Research Infrastructure. Twin Res Hum Genet. Dec 2019;22(6):672–680. doi:10.1017/thg.2019.99 31747977

[19] Leitsalu L, Haller T, Esko T, Cohort Profile: Estonian Biobank of the Estonian Genome Center, University of Tartu. Int J Epidemiol. Aug 2015;44(4):1137–47. doi:10.1093/ije/dyt268 24518929

[20] Ojalo T, Haan E, Koiv K, Cohort Profile Update: Mental Health Online Survey in the Estonian Biobank (EstBB MHoS). Int J Epidemiol. Feb 14 2024;53(2)doi:10.1093/ije/dyae017 PMC10881104 38381979

[21] Milani L, Alver M, Laur S, From Biobanking to Personalized Medicine: the journey of the Estonian Biobank. medRxiv. 2024:2024.09.22.24313964. doi:10.1101/2024.09.22.24313964

[22] Magnus P, Birke C, Vejrup K, Cohort Profile Update: The Norwegian Mother and Child Cohort Study (MoBa). Int J Epidemiol. Apr 2016;45(2):382–8. doi:10.1093/ije/dyw029 27063603

[23] Demontis D, Walters GB, Athanasiadis G, Genome-wide analyses of ADHD identify 27 risk loci, refine the genetic architecture and implicate several cognitive domains. Nat Genet. Feb 2023;55(2):198–208. doi:10.1038/s41588-022-01285-8 36702997 PMC10914347

[24] Strom NI, Verhulst B, Bacanu SA, Genome-wide association study of major anxiety disorders in 122,341 European-ancestry cases identifies 58 loci and highlights GABAergic signaling. medRxiv. Jul 5 2024;doi:10.1101/2024.07.03.24309466 PMC12900644 41634414

[25] Nievergelt CM, Maihofer AX, Atkinson EG, Genome-wide association analyses identify 95 risk loci and provide insights into the neurobiology of post-traumatic stress disorder. Nat Genet. May 2024;56(5):792–808. doi:10.1038/s41588-024-01707-9 38637617 PMC11396662

[26] O’Connell KS, Koromina M, van der Veen T, Genomics yields biological and phenotypic insights into bipolar disorder. Nature. Mar 2025;639(8056):968–975. doi:10.1038/s41586-024-08468-9 39843750 PMC12163093

[27] Schizophrenia Working Group of the Psychiatric Genomics C. Biological insights from 108 schizophrenia-associated genetic loci. Nature. Jul 24 2014;511(7510):421–7. doi:10.1038/nature13595 25056061 PMC4112379

[28] Lloyd-Jones LR, Zeng J, Sidorenko J, Improved polygenic prediction by Bayesian multiple regression on summary statistics. Nat Commun. Nov 8 2019;10(1):5086. doi:10.1038/s41467-019-12653-0 31704910 PMC6841727

[29] Choi SW, O’Reilly PF. PRSice-2: Polygenic Risk Score software for biobank-scale data. Gigascience. Jul 1 2019;8(7)doi:10.1093/gigascience/giz082 PMC6629542 31307061

[30] Bakken IJ, Ariansen AMS, Knudsen GP, Johansen KI, Vollset SE. The Norwegian Patient Registry and the Norwegian Registry for Primary Health Care: Research potential of two nationwide health-care registries. Scand J Public Health. Feb 2020;48(1):49–55. doi:10.1177/1403494819859737 31288711

[31] Lee SH, Goddard ME, Wray NR, Visscher PM. A better coefficient of determination for genetic profile analysis. Genet Epidemiol. Apr 2012;36(3):214–24. doi:10.1002/gepi.21614 22714935

[32] Bergstedt J, Pasman JA, Ma Z, Distinct biological signature and modifiable risk factors underlie the comorbidity between major depressive disorder and cardiovascular disease. Nat Cardiovasc Res. 2024;3(6):754–769. doi:10.1038/s44161-024-00488-y 39215135 PMC11182748

[33] Veeneman RR, Vermeulen JM, Abdellaoui A, Exploring the Relationship Between Schizophrenia and Cardiovascular Disease: A Genetic Correlation and Multivariable Mendelian Randomization Study. Schizophr Bull. Mar 1 2022;48(2):463–473. doi:10.1093/schbul/sbab132 34730178 PMC8886584

[34] Bahrami S, Steen NE, Shadrin A, Shared Genetic Loci Between Body Mass Index and Major Psychiatric Disorders: A Genome-wide Association Study. JAMA Psychiatry. May 1 2020;77(5):503–512. doi:10.1001/jamapsychiatry.2019.4188 31913414 PMC6990967

[35] Reponen EJ, Ueland T, Rokicki J, Polygenic risk for schizophrenia and bipolar disorder in relation to cardiovascular biomarkers. Eur Arch Psychiatry Clin Neurosci. Aug 2024;274(5):1223–1230. doi:10.1007/s00406-023-01591-0 37145175 PMC11226473

[36] Rodevand L, Rahman Z, Hindley GFL, Characterizing the Shared Genetic Underpinnings of Schizophrenia and Cardiovascular Disease Risk Factors. Am J Psychiatry. Nov 1 2023;180(11):815–826. doi:10.1176/appi.ajp.20220660 37752828 PMC11780279

[37] Alver M, Kasela S, Haring L, Genetic predisposition and antipsychotic treatment effect on metabolic syndrome in schizophrenia: a ten-year follow-up study using the Estonian Biobank. Lancet Reg Health Eur. Jun 2024;41:100914. doi:10.1016/j.lanepe.2024.100914 38707868 PMC11066665

[38] Milaneschi Y, Simmons WK, van Rossum EFC, Penninx BW. Depression and obesity: evidence of shared biological mechanisms. Mol Psychiatry. Jan 2019;24(1):18–33. doi:10.1038/s41380-018-0017-5 29453413

[39] Cortese S, Moreira-Maia CR, St Fleur D, Morcillo-Penalver C, Rohde LA, Faraone SV. Association Between ADHD and Obesity: A Systematic Review and Meta-Analysis. Am J Psychiatry. Jan 2016;173(1):34–43. doi:10.1176/appi.ajp.2015.15020266 26315982

[40] Pasman JA, Bergstedt J, Harder A, Causes and consequences of major depressive disorder: An encompassing Mendelian randomization study. medRxiv. 20240521 ed2024. 10.1038/s44220-025-00471-x PMC12417207 40932904

[41] Charach A, Yeung E, Climans T, Lillie E. Childhood attention-deficit/hyperactivity disorder and future substance use disorders: comparative meta-analyses. J Am Acad Child Adolesc Psychiatry. Jan 2011;50(1):9–21. doi:10.1016/j.jaac.2010.09.019 21156266

[42] Gurillo P, Jauhar S, Murray RM, MacCabe JH. Does tobacco use cause psychosis? Systematic review and met-aanalysis. Lancet Psychiatry. Aug 2015;2(8):718–725. doi:10.1016/S2215-0366(15)00152-2 26249303 PMC4698800

