# Supplementary material for: Association of Polygenic Risk for Psychiatric Disorders with Cardiometabolic Disease
Source: medRxiv. 2025 Mar 11:2025.03.11.25323757. Preprint. [Version 1] doi: 10.1101/2025.03.11.25323757 (PMC11952624; doi:10.1101/2025.03.11.25323757)
Supplement: Supplementary file 1 [file media-1.pdf]

eTable 1: Source GWASs for PRSs. ADHD, attention deficit/hyperactivity disorder; GWAS, genome-wide association study; MDD, major depressive disorder; PTSD, post-traumatic stress disorder.

Trait	Discovery GWAS	PMID	Prevalence	N case	N controls	h^2
ADHD	Demontis et al., 2023	36702997	0.05	38,691	275,986	0.22
Major depression	Als et al. 2023	37464041	0.15	294,322	741,438	0.07
Anxiety disorder	Purves et al., 2020	31748690	0.06	31,977	82,114	0.26
PTSD	Nievergelt et al., 2019	31594949	0.05	23,185	151,309	0.05
Bipolar disorder	Mullins et al., 2021	34002096	0.01	41,917	371,549	0.16
Schizophrenia	Trubetskoy et al., 2022	35396580	0.00	53,386	77,258	0.23

eTable 2: Number of variants for PRS derived using genome-wide significant variants. ADHD, attention deficit/hyperactivity disorder; MDD, major depressive disorder; PTSD, post-traumatic stress disorder; PRS, polygenic risk score; SNP, single nucleotide polymorphism.

Cohort	Trait	Number of SNPs
STR	ADHD	36
	MDD	189
	Anxiety disorder	3
	PTSD	1
	Bipolar disorder	78
	Schizophrenia	280
EstBB	ADHD	37
	MDD	226
	Anxiety disorder	3
	PTSD	1
	Bipolar disorder	72
	Schizophrenia	274
MoBa	ADHD	35
	MDD	233
	Anxiety disorder	3
	PTSD	1
	Bipolar disorder	79
	Schizophrenia	349

eTable 3: ICD-codes for psychiatric disorders. ADHD, attention deficit/hyperactivity disorder; ICD, international classification of diseases; MDD, major depressive disorder.

Disorder	ICD-8	ICD-9	ICD-10
ADHD		314	F90
MDD	296,2 300,4 298,0	296B 311 300E 298A	F32 F33 F341 F348 F349 F38 F39
Anxiety disorders	300,0 300,2	300A 300C	F40 F41
Stress-related disorders	307	308 309	F43
Bipolar disorder	296,1 296,3 296,8	296A 296C 296D 296E 296F 296G 296H 296W 296X	F30 F31
Schizophrenia	295	295	F20

eTable 4: ICD-codes for cardiometabolic diseases. ICD, international classification of diseases

Disease	ICD-8	ICD-9	ICD-10
Hyperlipidemia	279	272	E78
Obesity	277	278A 278B	E65 E66
Type 2 diabetes	250	250	E11
Hypertensive diseases	400 401 402 403 404	401 402 403 404 405	I1
Arteriosclerosis	440 441 442 443 444	440 441 442 443 444	I70 I71 I72 I73 I74
Ischemic heart diseases	410 411 412 413 414	410 411 412 413 414	I20 I21 I22 I23 I24 I251 I252 I255 I256 I258 I259
Heart failure	428	428	I50
Arrhythmias		426A 426B 427A 427B 427D 427E 427F 427W	I441 I442 I46 I470 I471 I472 I48 I490 I495 I498
Thromboembolic disease	450 451	415B 451B	I26 I80
Cerebrovascular diseases	43	43	I6 G45

eTable 5: Measures for predictive performance (e.g. different pseudo-R^2 measures) of PRSs for psychiatric disorders per cohort. Note that ADHD is missing for STR because of a lack of diagnosed cases in that cohort.
ADHD, attention deficit/hyperactivity disorder; EstBB, Estonian Biobank; MDD, major depressive disorder; PTSD, post-traumatic stress disorder; PRS, polygenic risk score; STR, Swedish Twin Registry.

Type	Value (%)	Cohort	Outcome	Exposure
R2 liability	0.40	STR	Anxiety disorder	Anxiety disorder PRS
R2 logit liability	0.66	STR	Anxiety disorder	Anxiety disorder PRS
Nagelkerke	0.27	STR	Anxiety disorder	Anxiety disorder PRS
R2 liability	7.19	STR	Bipolar disorder	Bipolar disorder PRS
R2 logit liability	14.45	STR	Bipolar disorder	Bipolar disorder PRS
Nagelkerke	4.91	STR	Bipolar disorder	Bipolar disorder PRS
R2 liability	1.33	STR	MDD	MDD PRS
R2 logit liability	1.88	STR	MDD	MDD PRS
Nagelkerke	0.91	STR	MDD	MDD PRS
R2 liability	9.88	STR	Schizophrenia	Schizophrenia PRS
R2 logit liability	23.90	STR	Schizophrenia	Schizophrenia PRS
Nagelkerke	7.03	STR	Schizophrenia	Schizophrenia PRS
R2 liability	0.42	STR	Stress-related disorders	PTSD PRS
R2 logit liability	0.77	STR	Stress-related disorders	PTSD PRS
Nagelkerke	0.28	STR	Stress-related disorders	PTSD PRS
R2 liability	1.34	EstBB	ADHD	ADHD PRS
R2 logit liability	3.02	EstBB	ADHD	ADHD PRS
Nagelkerke	0.91	EstBB	ADHD	ADHD PRS
R2 liability	0.89	EstBB	Anxiety disorder	Anxiety disorder PRS
R2 logit liability	0.81	EstBB	Anxiety disorder	Anxiety disorder PRS
Nagelkerke	0.70	EstBB	Anxiety disorder	Anxiety disorder PRS
R2 liability	1.75	EstBB	Bipolar disorder	Bipolar disorder PRS
R2 logit liability	3.99	EstBB	Bipolar disorder	Bipolar disorder PRS
Nagelkerke	1.19	EstBB	Bipolar disorder	Bipolar disorder PRS
R2 liability	2.48	EstBB	MDD	MDD PRS
R2 logit liability	2.14	EstBB	MDD	MDD PRS
Nagelkerke	2.00	EstBB	MDD	MDD PRS
R2 liability	3.23	EstBB	Schizophrenia	Schizophrenia PRS
R2 logit liability	11.73	EstBB	Schizophrenia	Schizophrenia PRS
Nagelkerke	3.40	EstBB	Schizophrenia	Schizophrenia PRS
R2 liability	0.17	EstBB	Stress-related disorders	PTSD PRS
R2 logit liability	0.20	EstBB	Stress-related disorders	PTSD PRS
Nagelkerke	0.13	EstBB	Stress-related disorders	PTSD PRS
R2 liability	3.38	MoBa	ADHD	ADHD PRS
R2 logit liability	6.07	MoBa	ADHD	ADHD PRS
Nagelkerke	2.27	MoBa	ADHD	ADHD PRS
R2 liability	1.15	MoBa	Anxiety disorder	Anxiety disorder PRS
R2 logit liability	1.51	MoBa	Anxiety disorder	Anxiety disorder PRS
Nagelkerke	0.79	MoBa	Anxiety disorder	Anxiety disorder PRS
R2 liability	3.18	MoBa	Bipolar disorder	Bipolar disorder PRS
R2 logit liability	6.45	MoBa	Bipolar disorder	Bipolar disorder PRS
Nagelkerke	2.15	MoBa	Bipolar disorder	Bipolar disorder PRS
R2 liability	3.77	MoBa	MDD	MDD PRS
R2 logit liability	4.49	MoBa	MDD	MDD PRS
Nagelkerke	2.66	MoBa	MDD	MDD PRS
R2 liability	4.27	MoBa	Schizophrenia	Schizophrenia PRS
R2 logit liability	13.75	MoBa	Schizophrenia	Schizophrenia PRS
Nagelkerke	3.13	MoBa	Schizophrenia	Schizophrenia PRS
R2 liability	0.45	MoBa	Stress-related disorders	PTSD PRS
R2 logit liability	0.56	MoBa	Stress-related disorders	PTSD PRS
Nagelkerke	0.32	MoBa	Stress-related disorders	PTSD PRS

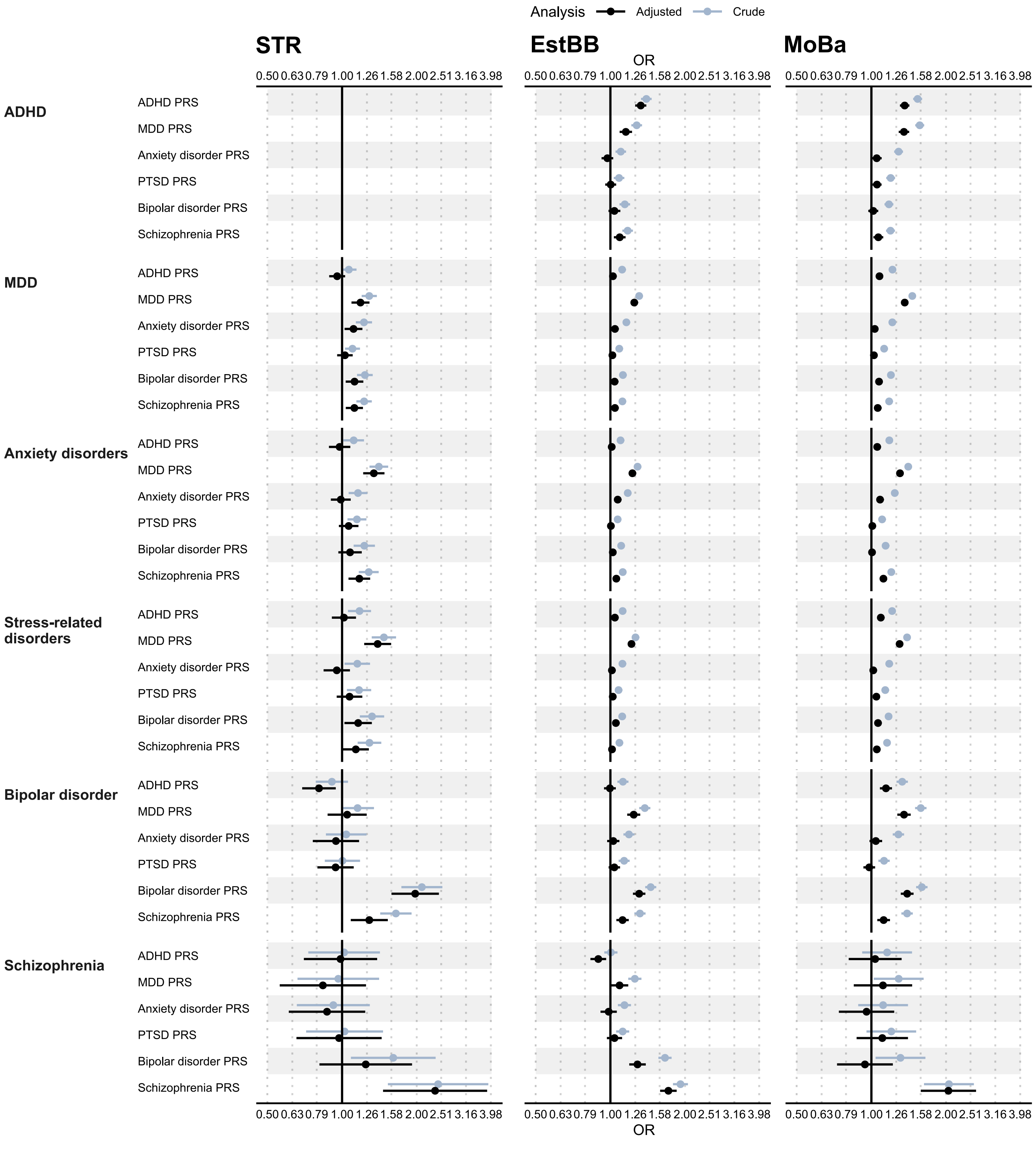

eFigure 1. Associations of psychiatric PRSs with clinical diagnoses of psychiatric disorders in STR, EstBB, and MoBa. ORs were computed using logistic regression models with the PRS as exposure variable and any recorded clinical diagnosis of the psychiatric disorder as outcome variable. The crude model adjusted for principal components, sex, and age. The adjusted model additionally adjusted for all psychiatric PRSs. ADHD, attention deficit/hyperactivity disorder; AOR, adjusted odds ratio; MDD, major depressive disorder; OR, odds ratio; PTSD, post-traumatic stress disorder; PRS, polygenic risk score.

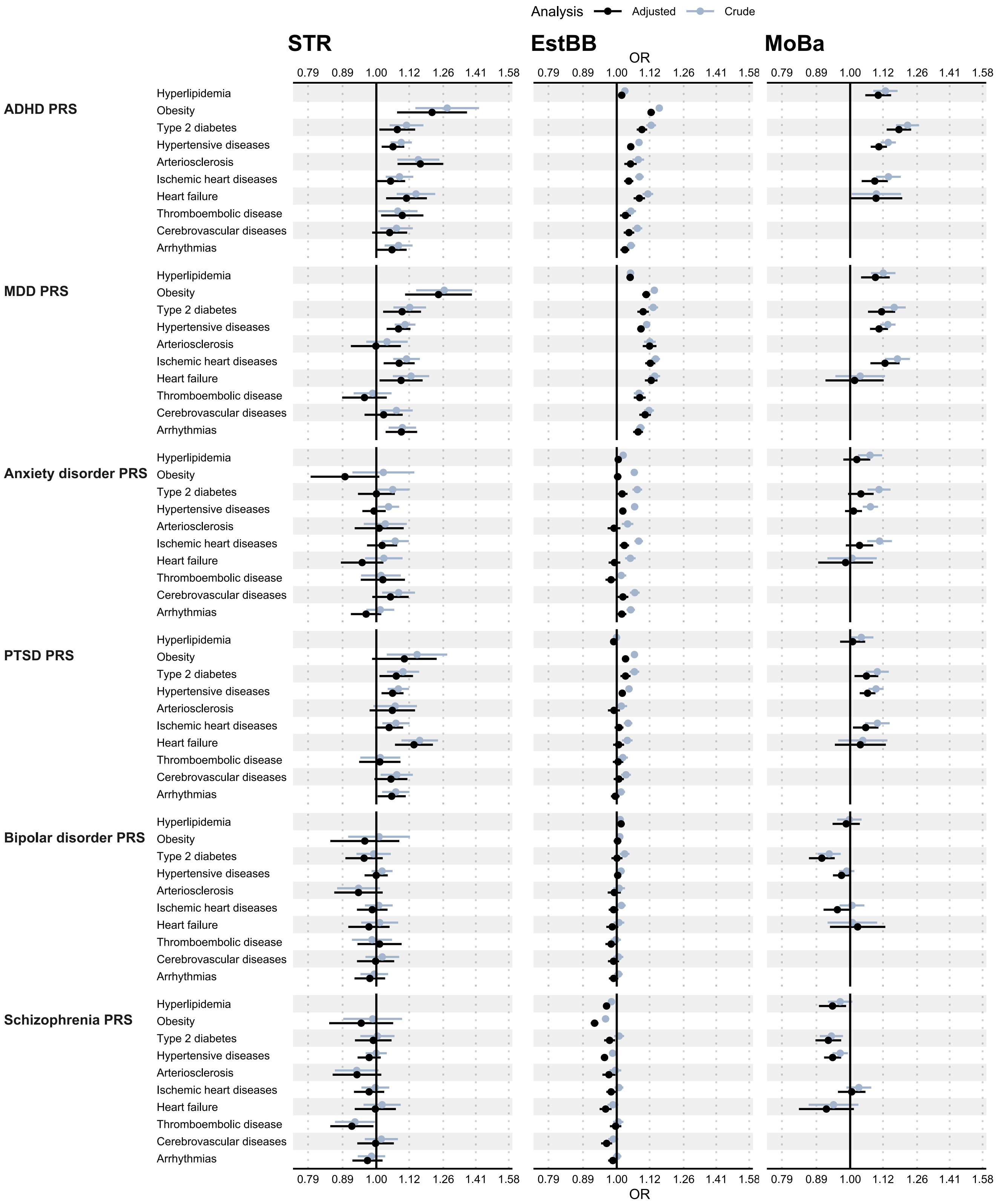

eFigure 2. Associations of psychiatric PRSs with clinical diagnoses of CMDs in STR, EstBB, and MoBa. ORs were computed using logistic regression models with the PRS as exposure variable and any recorded clinical diagnosis of the CMD as outcome variable. The crude model adjusted for principal components, sex, and age. The adjusted model additionally adjusted for all psychiatric PRSs. ADHD, attention deficit/hyperactivity disorder; AOR, adjusted odds ratio; CMD, cardiometabolic disease. MDD, major depressive disorder; OR, odds ratio; PTSD, post-traumatic stress disorder; PRS, polygenic risk score.

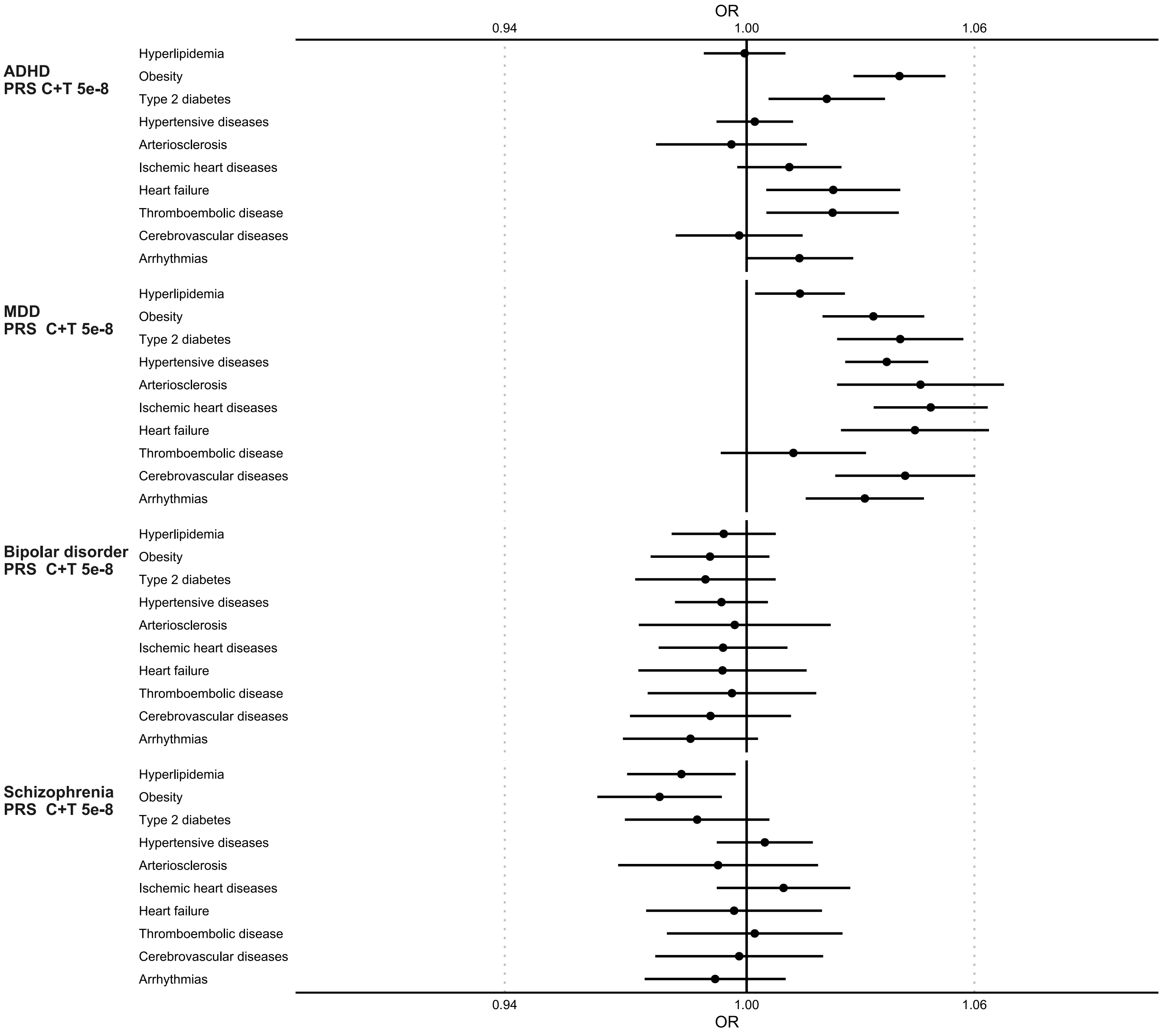

eFigure 3. Associations of psychiatric PRSs derived using genome-wide significant variants with clinical diagnoses of CMDs. ORs were computed using logistic regression models with the PRS as exposure variable and any recorded clinical diagnosis of the CMD as the outcome variable. The model adjusted for principal components, sex, and age. Results for anxiety disorder and PTSD are not shown because to few variants reached the genome-wide significance threshold (<4). PRSs were derived using clumping and thresholding on P < 5e-8. ORs were computed by meta-analyzing ORs in STR, EstBB, and MoBa using fixed effects meta-analysis model. ADHD, attention deficit/hyperactivity disorder; CMD, cardiometabolic disease; C+T, clumping plus thresholding; MDD, major depressive disorder; OR, odds ratio; PTSD, post-traumatic stress disorder; PRS, polygenic risk score.

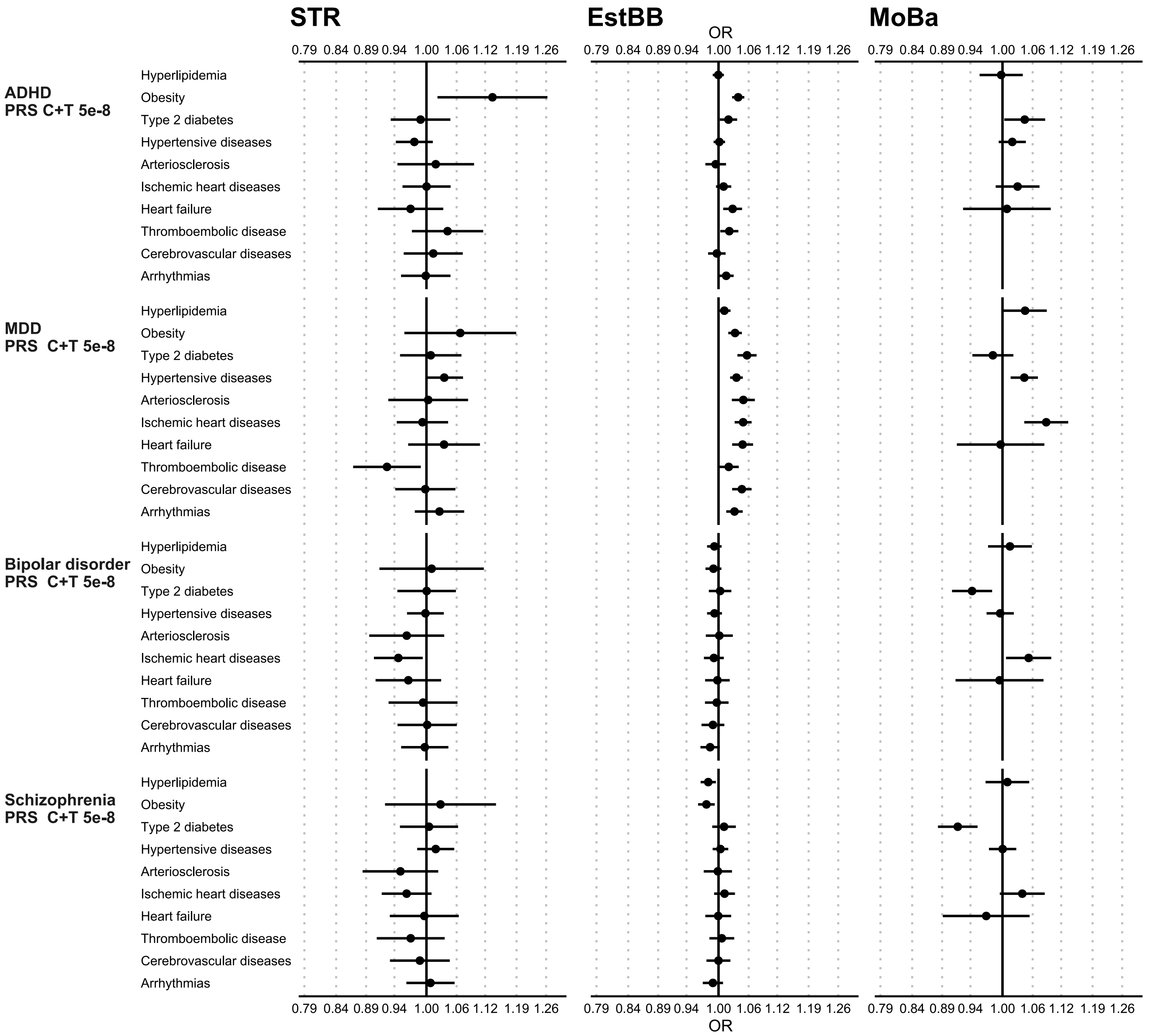

eFigure 4. Associations of psychiatric PRSs derived using genome-wide significant variants with clinical diagnoses of CMDs in three cohorts. ORs were computed using logistic regression models with the PRS as exposure variable and any recorded clinical diagnosis of the CMD as the outcome variable. The model adjusted for principal components, sex, and age. Results for anxiety disorder and PTSD are not shown because to few variants reached the genome-wide significance threshold (<4). PRSs were derived using clumping and thresholding on P < 5e-8. ADHD, attention deficit/hyperactivity disorder; CMD, cardiometabolic disease; C+T, clumping plus thresholding; MDD, major depressive disorder; OR, odds ratio; PTSD, post-traumatic stress disorder; PRS, polygenic risk score.

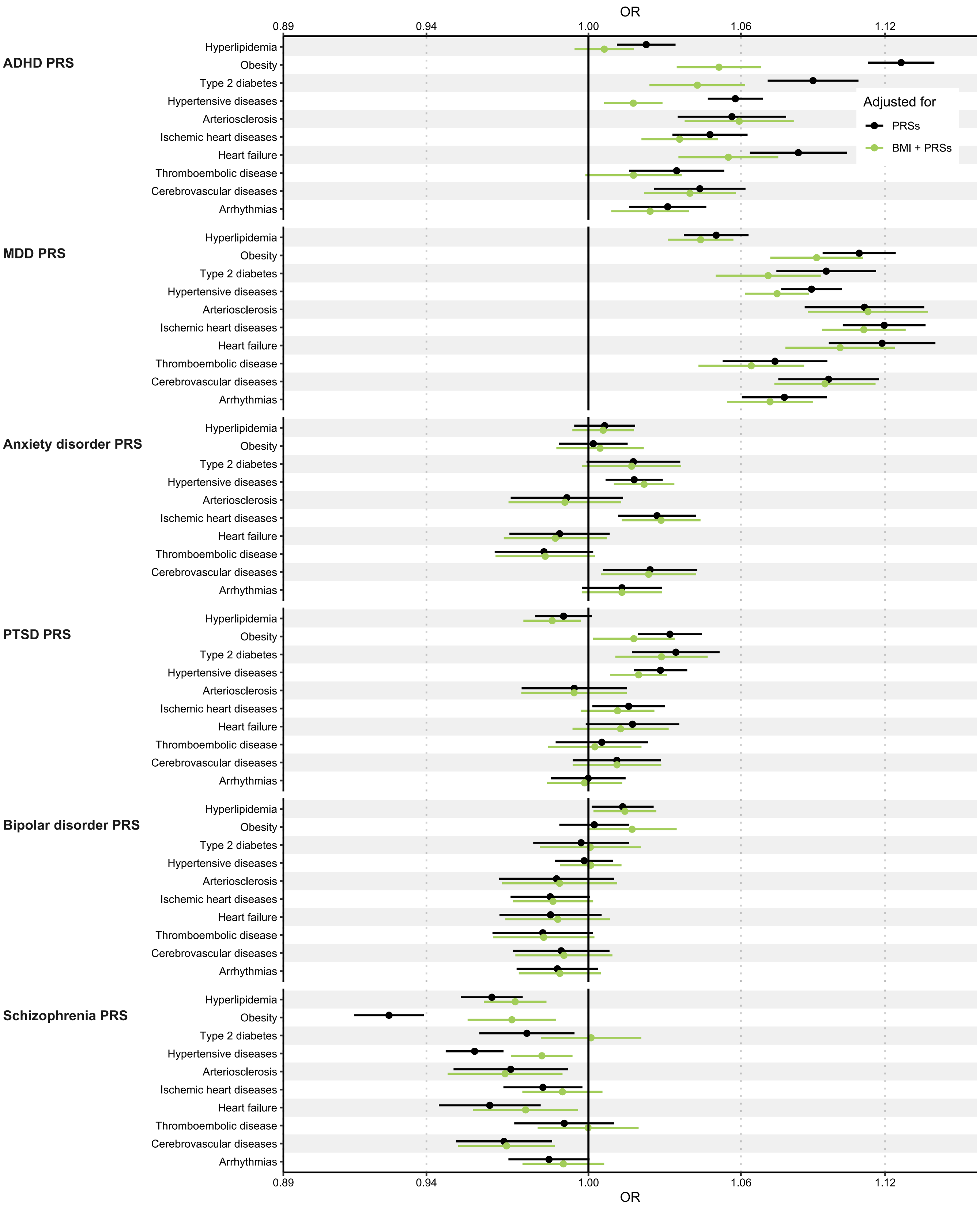

eFigure 5. Associations of psychiatric PRSs with clinically diagnosed CMDs adjusting for BMI. ORs were computed using logistic regression models with the PRS as exposure variable and any recorded clinical diagnosis of the CMD as outcome variable and other psychiatric PRSs, sex, birth-year, and 10 principal components as covariates. The "BMI + PRSs" model additionally adjusted for BMI levels. ORs were computed by meta-analyzing ORs in STR, EstBB, and MoBa using a fixed effects meta-analysis model. ADHD, attention deficit/hyperactivity disorder; BMI, body mass index; CMD, cardiometabolic disease; MDD, major depressive disorder; OR, odds ratio; PTSD, post-traumatic stress disorder; PRS, polygenic risk score.
